# Lesion-specific features of macrophage polarization contribute to *Mycobacterium tuberculosis* infection in the lungs of patients with pulmonary tuberculosis

**DOI:** 10.3389/fimmu.2026.1853352

**Published:** 2026-06-12

**Authors:** Elena G. Ufimtseva, Natalya I. Eremeeva, Sergey N. Skornyakov

**Affiliations:** 1 Laboratory of Medical Biotechnology, Institute of Biochemistry, Federal Research Center of Fundamental and Translational Medicine, Novosibirsk, Russia; 2 Institute of Disinfectology, F.F. Erisman Federal Scientific Center of Hygiene of the Federal Service on Surveillance for Consumer Rights Protection and Human Well-being, Moscow, Russia; 3 Scientific Department, Ural Research Institute for Phthisiopulmonology, National Medical Research Center of Tuberculosis and Infectious Diseases of Ministry of Health of the Russian Federation, Yekaterinburg, Russia

**Keywords:** AhR–AhRR–CYP1A1 axis, cytokines, fibrosis, mixed M1/M2 polarization, *Mycobacterium tuberculosis*, NF-κB pathway, pulmonary tuberculosis, smoker’s alveolar macrophages

## Abstract

**Background:**

The innate immunity, linked to macrophage polarization, plays a critical role in the progression of tuberculosis (TB) disease caused by *M. tuberculosis* (*Mtb*) infection. Within a single patient with active pulmonary TB, *Mtb* infection leads to the formation of extensive lung pathology with a spectrum of morphologically and physiologically distinct TB lesions at different stages of progression, where macrophages are believed to represent varying functional phenotypes. Understanding the dynamics of macrophage polarization with identification of the cytokine network, molecular pathways, and polarizing stimuli in the lung TB lesions of patients, depending on various aspects of the host-pathogen interactions, is crucial for developing better treatment strategies, based on immune-modulatory therapeutic approaches, especially in the context of the ongoing spread of drug-resistant TB.

**Methods:**

The expression of pro-inflammatory/anti-bacterial M1 cytokines (IFNγ, TNFα, IL-1β, and IL-12) and anti-inflammatory/immunosuppressive M2 cytokines (IL-4, IL-10, and FGF2) as well as activation of the AhR and NF-*κ*B pathways were estimated in an immunofluorescence assay and analyzed in relation to *Mtb* infection in macrophages in the *ex vivo* cell cultures and/or on the histological sections obtained from various lung TB lesions of the same patients (*n* = 25) with clinically active pulmonary, predominantly drug-resistant TB.

**Results:**

Macrophage polarization was determined by local tissue microenvironments with varying fibrosis severity but was independent of *Mtb*’s genetic and phenotypic diversity in human lungs, the anti-TB treatment of patients before surgery and AhR signaling activity in smoker’s macrophages. Pathogen control (without complete *Mtb* clearance) correlated with co-expression of cytokines of both cytokine types in the mixed M1/M2-polarized macrophages and, presumably, with NF-*κ*B-mediated activation of smoker’s alveolar macrophages in the lung TB lesions with local/minimal fibrosis and the preserved alveoli. In contrast, suppressed macrophages (lacking cytokine production and NF-*κ*B-activated mediators and, in parallel, harboring higher *Mtb* loads) exhibited an M0-like polarization state in tuberculoma walls and extensively fibrotic lung tissues.

**Conclusions:**

The host cells’ inability to eradicate *Mtb* reflected suboptimal development of the immune responses in the patients’ lungs. Defining the complexity and functional diversity of macrophage phenotypes and signaling pathways involved in the pathogen control and tissue pathology across various lung TB lesions within the same patients with pulmonary TB is essential for designing novel host-directed therapies that modulate the patients’ immune responses to optimize protection against *Mtb* infection and prevent the pathogen transmission.

## 1 Introduction

Tuberculosis (TB) is a serious disease caused by *Mycobacterium tuberculosis* (*Mtb*) infection, and it remains one of the major causes of infectious disease mortality worldwide (1–3). The occurrence, progression and regression of TB disease in human lungs correlate not only with the virulence and invasiveness of *Mtb*, but also with the immunity of patients, when the lung-resident macrophages, mostly alveolar macrophages, provide the first line of the host’s defense against the pathogen (2, 4–6). Macrophages not only possess *Mtb* recognition, engulfment, and killing functions, but also produce soluble mediators and cytokines that allow them to participate in different stages of the inflammatory reaction, from the initiation to the resolution phase (2, 4–9). Paradoxically, these cells are the dominant ecological niche for *Mtb* survival and replication in phagosomes, as a result of the inhibition of phagosome maturation during human TB (2, 10–12).

The general concept, largely based on *in vitro* culture systems and studies in mice (13–15), is that during TB infection, naïve alveolar macrophages, which typically do not express any cytokines or active molecules (M0 polarization state), differentiate into phenotypically and functionally polarized subsets, mainly consisting of the classically activated M1 macrophages and the alternatively activated M2 macrophages, which have pro-inflammatory and anti-inflammatory phenotypes, respectively (7, 13–17). Macrophages in the M1-polarized state exhibit the pathogen elimination and pro-inflammatory program through high expression of pro-inflammatory and anti-microbial molecules such as interferon gamma (IFNγ), tumor necrosis factor alpha (TNFα), interleukin-1 beta (IL-1β), interleukin-12 (IL-12), and the release of reactive oxygen species (ROS) and nitrogen radicals, induced by the activation of inducible nitric oxide synthase (iNOS), to kill *Mtb* (6, 8, 12, 18). IFNγ and TNFα are the key cytokines for controlling *Mtb* infection, and their deficiency can lead to uncontrolled infection, while their excessive production can cause immunopathology, the formation of caseous necrosis, and considerable lung damage due to fibrotic processes (18–22). In contrast, the M2-polarized macrophages prevent overactivation of the immune system and control the resolution of inflammation by repairing and remodeling the damaged lung tissue, but may also antagonize the anti-bacterial effects and support *Mtb* growth by expressing high levels of anti-inflammatory cytokines such as interleukin-4 (IL-4) and interleukin-10 (IL-10) (13–18).

At early stages of *Mtb* infection in human lungs, the polarization state of macrophages is assumed to be mainly of the M1 type, while at later stages of infection and during active TB disease, macrophage polarization is dominated by the M2 type (2, 7–9, 16–18). However, to date, the progression of the macrophage subtypes in human TB has not been fully characterized. Moreover, observations of various human pathologies indicate that the dichotomous M1/M2 paradigm is an oversimplification and insufficient to describe the wide range of physiologically relevant macrophage states with often overlapping features and phenotypes that occur as a result of a dynamic interplay among a plethora of signals and stimuli present in the local environment and dependent on the context of complex clinical conditions (23–25). Nonetheless, studying the M1 and M2 macrophage phenotypes and their overlaps is considered to be very useful for understanding various immunopathological reactions in human lungs following *Mtb* infection (7, 23–25). Their regulation holds promise as a new therapeutic approach, the so-called host-directed therapy (HDT), for TB patients in clinical settings (26–28), especially in the context of the ongoing spread of drug-resistant TB (1–3).

Since multiple layers of control govern macrophage development, activation, and function in human lungs during TB infection (29, 30), reprogramming of macrophage phenotypes by use of various cytokines and drugs is one of the main HDT strategies at improving TB treatment outcomes (19, 26–30). At the same time, within a single TB patient, *Mtb* infection leads to the formation of extensive lung pathology with a spectrum of morphologically and physiologically distinct TB lesions at different stages of progression (31–33). The lung macrophages are believed to represent distinct functional phenotypes in various TB lesions (34, 35), hence HDT may be effective only in certain lung lesion types (36–38). While fundamental determinants of the granuloma structure and pathogenesis, such as the composition and distribution of immune cells and their expression patterns of CD molecules, cytokine receptors, and some other markers, have been identified using multiplexed immunofluorescence on histological sections from the lungs of patients with active TB disease (39–42), the *Mtb* loads, as well as the production of most pro-inflammatory and anti-inflammatory cytokines and molecules by macrophages, were not evaluated in these studies. However, these parameters form the basis for macrophage-centered therapeutic strategies in HDT to prevent and treat human TB (36–38, 43).

For a better understanding of TB dynamics in the context of host-pathogen relationships and their regulation, we have developed a technique to obtain macrophages from lung tissue surgically removed from patients with pulmonary TB after intensive anti-TB chemotherapy (10). We found variations in *Mtb* loads across the patients’ lung TB lesions, as well as different subpopulations of multidrug-tolerant *Mtb* with a spectrum of phenotypic and growth characteristics in the same TB lesions (10, 11, 44), when non-acid-fast *Mtb* expressing the universal stress protein domain-containing protein Rv2623, but lacking virulence factors such as lipoarabinomannan (LAM) and 6-kDa early secretory antigenic target protein (ESAT-6) accounted for about one-third of each bacterial population examined (45). However, no regulatory mechanisms linked to macrophage cytokine signaling were characterized in the pathological areas examined.

In this work, studying the expression of pro-inflammatory/anti-bacterial M1 cytokines (IFNγ, TNFα, IL-1β, IL-12) and anti-inflammatory/immunosuppressive M2 cytokines (IL-4, IL-10, fibroblast growth factor 2 (FGF2)) and its regulatory mechanisms, connecting with the transcription factor aryl hydrocarbon receptor (AhR) and nuclear factor kappa-light-chain-enhancer of activated B cells (NF-*κ*B) pathways, in the bactericidal activation of macrophages identified the lesion-specific features of their polarization and signaling in the lungs of patients with pulmonary TB. Smoker’s (i.e., alveolar) macrophages, which were the main subset of cells in the macrophage populations for each lung tissue sample, demonstrated activation of the AhR pathway in all lung TB lesions examined. However, bacterial control (though not complete *Mtb* clearance) was associated, presumably, only with the NF-*κ*B pathway-induced inflammation and co-expression of pro- and anti-inflammatory cytokines in alveolar macrophages, reflecting a mixed M1/M2 polarization state in the lung regions with multiple alveoli. For the same patients, a majority of macrophages in tuberculoma walls and the lung tissues with the absence of alveoli and extensive fibrosis exhibited an M0-like polarization state characterized by a lack of cytokine production, probably as a result of inhibiting NF-*κ*B-mediated processes, and presented significantly higher *Mtb* loads within cells. These findings demonstrate the complexity and functional diversity of human macrophage phenotypes and signaling pathways across local lung lesions within the same TB patients and should be taken into account for the successful development of new immune strategies and HDT regimens to optimize protection against *Mtb* infection and improve pathology control.

## 2 Materials and methods

### 2.1 Patients and lung tissue samples

Lung tissue specimens were obtained from 25 patients with clinically active pulmonary TB who had undergone surgical resection of infected tissue due to disease severity and treatment failure at the Department of Thoracic Surgery of the Ural Research Institute for Phthisiopulmonology (Yekaterinburg, Russia) affiliated with the National Medical Research Center of Tuberculosis and Infectious Diseases of the Ministry of Health of the Russian Federation (Moscow, Russia) [see (10, 11, 46)]. The patients had received supervised TB treatment through their local clinics. Detailed patient characteristics (age, gender, smoking status, treatment, comorbidities, surgery, etc.) and the pathogen (genotype family, drug-resistance mutations in *Mtb* genes, virulence in the guinea pig TB model, etc.) were previously reported [see (10, 11, 45, 46)] and some of them are presented here (**Supplementary Table 1**). For control purposes, blood samples were obtained from two healthy 20-year-old non-smoking volunteers, without TB or other diseases. The patients and healthy volunteers were residents of the Ural District of the Russian Federation. All TB patients and healthy volunteers provided written informed consent for the collection of clinical data, tissue collection, and research testing, approved by the Ethics Committees of the Ural Research Institute for Phthisiopulmonology affiliated with the National Medical Research Center of Tuberculosis and Infectious Diseases (Protocol No. 27/2014/07/02). Human studies were conducted in accordance with the Declaration of Helsinki. All patients included in the study had been referred for surgical management of severe pulmonary TB and thus had extensive tissue damage, including tuberculomas, fibrotic lesions, and caseous TB lesions in the lungs. Patients 10, 22, and 23 presented with cavities in the lungs. Immediately after surgery, lung tissue specimens (~ 0.5–30 g) were collected from areas approximately 5 cm away from macroscopic TB lesions, such as tuberculomas and cavities, for patients 1–5 and 10–29 (‘distant’ lung tissues/parts/tissue samples throughout), while pieces of tuberculoma walls (‘tuberculoma’ throughout) were collected only for patients 22–29 (**Supplementary Table 1**). All patients, except patient 10, were sputum smear-negative. The *Mtb* clinical isolates were obtained only from the lung tissue homogenates of patients 10, 11, and 20 [see (10, 45)].

### 2.2 *Ex vivo* isolation and culture of human cells

Alveolar macrophages were isolated from the specimens of surgically resected lung tissues of patients 1–5 and 10–29 (**Supplementary Table 1**) using methods described earlier [see (10, 11, 44, 46)]. All lung samples used for cell isolation contained large amounts of fibrotic tissue with small embedded TB granulomas. In brief, these samples were cut into small pieces and passed through a metal sieve with pore sizes of 0.5-2.0 mm in diameter in phosphate-buffer saline (PBS, pH 7.4) to separate the cell suspension containing macrophages from fibrotic tissue and small necrotic nodules/granulomas up to 5 mm in diameter, which were retained in the sieve and subsequently discarded. Cell pellets were centrifuged at 400 g for 5 minutes at room temperature and plated in 24-well plates (Orange Scientific, Belgium) containing cover glasses (~8 × 8 mm in size) placed in the bottom. Cells were cultured for 16–18 hours in 0.5 ml of RPMI 1640 complete growth medium supplemented with 10% fetal bovine serum, 2 mM glutamine and 50 µg/ml gentamicin (BioloT, Russia) at +37 °C in an atmosphere containing 5% CO_2_. In parallel, after centrifugation, cells from blood samples of healthy volunteers were also plated in 24-well plates under the same conditions.

After 16–18 hours of *ex vivo* culture, the growth medium containing dead cell debris was removed, and monolayer cultures of human cells on the coverslips were washed twice with PBS to remove non-adherent cells. At this time point, in all the *ex vivo* cell cultures, over 90% of cells obtained from both tuberculoma walls and distant lung tissues from all patients were identified as macrophages [see (10, 11, 44, 46)]. In addition to macrophages, five other cell types were observed: dendritic cells, neutrophils, lymphocytes, fibroblasts, and multinucleate Langhans giant cells. However, these cell populations were present in significantly lower numbers [see (10, 44)]. Monolayer cultures derived from blood samples of healthy volunteers consisted predominantly of neutrophils and some monocytes, none of which contained cytoplasmic inclusions. Although TB patients had received intensive anti-TB drug treatment during the preoperative period (**Supplementary Table 1**), all cells – whether infected with *Mtb* or not – remained viable and showed no apoptotic or necrotic morphology in the *ex vivo* cell cultures [see (10, 11, 44–47)].

### 2.3 Cell staining

At 16–18 hours of *ex vivo* culture, after removal of growth medium with dead cell debris, monolayer cultures of cells on coverslips were washed with PBS and fixed with 4% formaldehyde in PBS for 10 minutes at room temperature. To visualize acid-fast *Mtb* within host cells, after washing with PBS, some cell preparations were stained by the Ziehl–Neelsen (ZN) method. After ZN staining, the cells were further counterstained with Mayer’s hematoxylin or a mixture of azure, eosin, and methylene blue in Romanowsky–Giemsa stain (Minimed, Moscow, Russia) according to the manufacturer’s instruction.

The other cell cultures were used for staining with antibodies and other reagents. To visualize *Mtb*, cytokines and other markers within cells, some cell preparations were washed with PBS, permeabilized with 0.3% Triton X-100 in PBS for 2 minutes, blocked in PBS containing 2% BSA, and incubated first with the appropriate primary antibodies: rabbit polyclonal antibodies to *Mycobacteria* LAM (Abcam, UK, ab20832) diluted 1:200, *Mtb* ESAT-6 (courtesy of E.V. Deineko of the Institute of Cytology and Genetics, SB RAS, Novosibirsk, Russia) diluted 1:300, human cyclooxygenase 2 (COX-2; Santa Cruz, USA, sc-1747-R) diluted 1:50, TNFα (Thermo Fisher Scientific, USA, P300A), IL-4 (Thermo Fisher Scientific, USA, PA5-25165), diluted 1:100 each, and mouse monoclonal antibodies to *Mtb* 38-kDa protein (Ag38; clone HTM81, Abcam, UK, ab183165) diluted 1:1000, human IFNγ (clone 25718, Thermo Fisher Scientific, USA, MA5-23718), IL-1β (clone 2805, Thermo Fisher Scientific, USA, MA5-23691), IL-10 (clone 945A2A5, Invitrogen, USA, AHC9102), IL-12p70 (clone 24945, Thermo Fisher Scientific, USA, MA5-23715), FGF2 (clone 6/basic FGF, BD Biosciences, USA, 610072), diluted 1:100 each, rat monoclonal PE-labeled antibodies to human IL-10 (clone JES3-9D7, eBioscience, USA, 12-7108-41) diluted 1:200, – and Alexa 488-labeled phalloidin dye (Invitrogen, USA, A12379) to stain filamentous actin (F-actin), diluted 1:200.

For all cell preparations, fluorescent visualization of bound primary antibodies was achieved using goat polyclonal secondary antibodies to rabbit IgG conjugated with DyLight 488 or DyLight 594 (Thermo Fisher Scientific, USA, 35553 or 35561, respectively), to mouse IgG conjugated with Alexa 488 or Alexa 555 (Thermo Fisher Scientific, USA, A-11001 or A-21422, respectively), and to rat IgG conjugated with Alexa 594 (Abcam, UK, ab150160) diluted 1:400 each. The cell preparations were incubated with the appropriate antibodies for 60 minutes at room temperature. Fluorescent staining was analyzed using the ProLong Gold Antifade Mountant with DAPI (Thermo Fisher Scientific, USA, P36935).

To control the reagents and confirm that the observed staining patterns are correct and reliable, the cell preparations obtained from various lung TB lesions (tuberculoma walls and distant lung tissues) of many patients (usually from 4 to 8 patients) and blood samples of healthy volunteers were simultaneously stained by different primary and secondary antibodies in various combinations.

### 2.4 Histology

The histological sections of the resected lung tissues of the patients were prepared as described [see (47, 48)]. In brief, the resected lung parts of the patients were cut into pieces. One portion of lung pieces was collected for producing alveolar macrophages as described above. The other portion of lung pieces was fixed with 4% formaldehyde in PBS (pH 7.4) for 20 hours at +4 °C. After fixation, the lung tissues were washed with PBS, incubated with 30% sucrose in PBS (pH 7.4) for 20 hours at +4 °C, frozen in Tissue-Tek O.C.T. Compound (Sakura Finetek, USA, 4583) at -25 °C, and sectioned at 16 µm on a Microtome Cryostat HM550 (Microm, Germany) at the Shared Center for Microscopic Analysis of Biological Objects of the Institute of Cytology and Genetics, SB RAS (Novosibirsk, Russia). Sections were air-dried on SuperFrost Plus slides (Thermo Fisher Scientific, USA). After washing with PBS, some preparations were stained with hematoxylin & eosin or by the ZN method. After ZN staining, the cells were further counterstained with a mixture of azure, eosin, and methylene blue in Romanowsky–Giemsa stain or Mayer’s hematoxylin (Minimed, Moscow, Russia) according to the manufacturer’s instruction.

Some of the histological sections were permeabilized with 0.3% Triton X-100 in PBS for 45 minutes, blocked, and incubated with the appropriate primary antibodies to the *Mtb* antigens, as described above, including mouse monoclonal antibodies to *Mtb* Rv2623 (clone A10, Abcam, UK, ab24291) diluted 1:1000 and human AhR (clone RPT9, Abcam, England, ab2769) diluted 1:100, human cytokines, as described above, and rabbit polyclonal antibodies to human AhR repressor (AhRR; Abcam, England, ab234817), cytochrome P450 family 1 subfamily A polypeptide 1 (CYP1A1; MyBioSource, USA, MBS178240), diluted 1:100 each, overnight at +4 °C in a humidity chamber and with the appropriate secondary antibodies for 60 minutes at room temperature. Fluorescent staining was analyzed using ProLong Gold Antifade Mountant with DAPI as described above.

The controls in the immunofluorescent staining experiments with the histological sections were made using the same rules as described above for the cell preparations.

### 2.5 Microscopy

The cytological and histological preparations were examined under an LSM 780 laser scanning confocal microscope (Zeiss, Germany) using the ZEN 2010 software (Zeiss, Germany) at the Shared Center for Microscopic Analysis of Biological Objects of the Institute of Cytology and Genetics, SB RAS (Novosibirsk, Russia). All the cytological preparations were analyzed for the expression of different cytokines in macrophages with or without *Mtb* in them using the ImageJ software. The cells were identified as the marker-positive when a two-fold or more increase in the intensity of the immunofluorescent staining of the marker in the cytoplasm of macrophages as compared to the background staining was detected and more than 30% of the cell surface was stained. All macrophages and leukocytes (more than 1000 cells for each marker staining: usually about 4000 cells for each marker) were analyzed in each cytological or histological preparation for each patient and each healthy volunteer, respectively. For the histological preparations, three non-serial tissue sections (~7 × 7 mm in size for each tissue section) from each individual sample were analyzed for each patient.

Confocal 3D merged fluorescent images of host cells, which were created to estimate the amount and morphology of *Mtb* in the cells [see (10)] with or without the cytokine expression in them, are mainly used in the Figures. It should be noted that sometimes these 3D images do not clearly demonstrate the accumulation of cytokines in host cells expressing cytokines, because they are focused on demonstrating the characteristics of the pathogen, whereas the cytokine expression in these cells was estimated with using confocal 2D fluorescent images. In addition, phase contrasted confocal 2D merged fluorescent images of the same cells were obtained to confirm the localization of *Mtb* in the cytoplasm of host cells both in the *ex vivo* cell cultures (**Supplementary Figure 1**) and on the histological sections [see (49)], but this type of images is not shown in the Figures.

### 2.6 Statistical analyses

Statistical data processing was performed using Prism 6.0 (GraphPad Software) and Microsoft Excel 2010 with each statistical test, definitions of mean ± standard error of the mean (SEM). The number of samples per group (*n*) was indicated in the corresponding text, figure legend, and panel. Because data in the compared groups did not significantly deviate from normality according to the Shapiro–Wilk test (all *P* > 0.05), and no heterogeneity of variances was detected by Levene’s test (*P* > 0.05), statistical significance for the comparisons between the datasets was determined using the independent-samples Student’s *t*-test. Differences were considered statistically significant at *P* < 0.05.

## 3 Results

### 3.1 No matter what pathogen characteristics, the macrophages with M1 as well as M2 cytokine expression are detected within the same cell populations in the majority of TB patients studied

**Figure 1:**
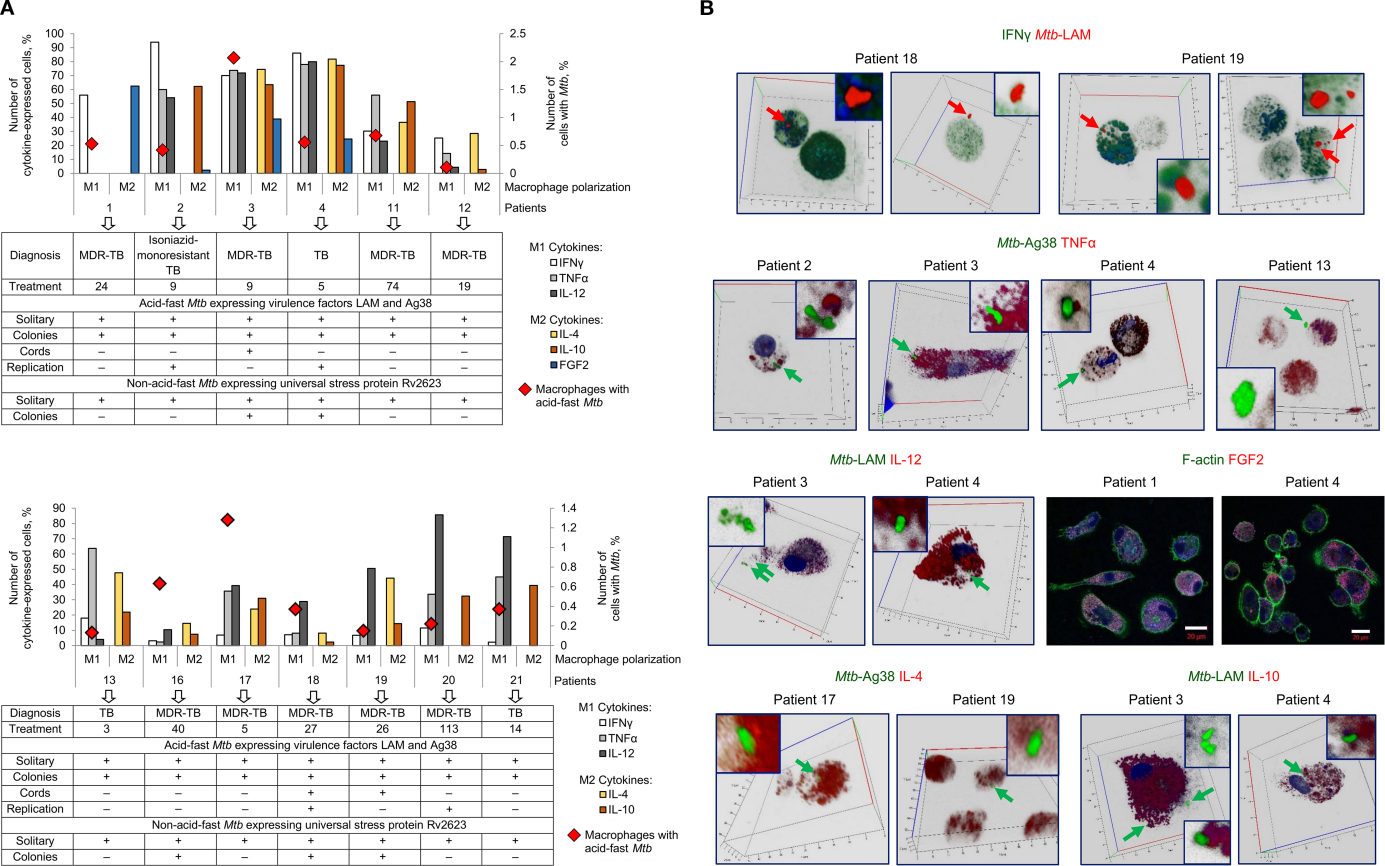
Regardless of the pathogen characteristics, macrophages with the M1 cytokines expression as well as macrophages producing M2 cytokines, with or without *Mtb* in them, are determined in the same cell populations obtained from the distant lung tissue samples of TB patients and analyzed after *ex vivo* culture for 16–18 hours. **(A)** The number of macrophages with the cytokines or acid-fast *Mtb* (solitary or as colonies) in them expressed as the percentage of the total number of the patients’ macrophages analyzed. The tables below the graphs show some characteristics of the patients and pathogen: (+), yes (–);, no. Anti-TB treatment in the months. **(B)** Representative confocal 3D and 2D merged fluorescent images of the macrophages stained by antibodies reacting with human cytokines and *Mtb* antigens (green or red signals) are shown. Nuclei are stained by DAPI (blue signal). Single and double arrows (green or red) point to *Mtb*, solitary or as colonies, and *Mtb* in colony with cording morphology, respectively. Close-ups of the parts of 3D images with *Mtb* are shown in the upper and/or lower corners. The scale bars are 20 μm each.

To understand the relationship between the pathogen characteristics and cytokine signaling in the regulation of bacterial clearance or persistence, the cytokine profiles were analyzed for the *Mtb* burden in the macrophages obtained from the distant lung tissue samples of patients 1–4, 11–13, and 16–21 (*n* = 13) after prolonged antibiotic therapy and simultaneously staining one of the virulence factors (LAM or Ag38) of *Mtb* and one of the M1- or M2-associated cytokines (IFNγ, TNFα, IL-12 or IL-4, IL-10, FGF2, respectively) in various combinations after *ex vivo* culture for 16–18 hours (**Figures 1A, B**). The characteristics of the patients (diagnosis, mainly multidrug-resistant TB (MDR-TB), and the duration of anti-TB treatment before surgery) and the pathogen (the *Mtb* loads with growth states of the pathogen, including the capability to replication in the prolonged *ex vivo* cell cultures under antibiotic-free conditions (‘replication’ throughout) and the formation of colonies with cording morphology (‘cords’ throughout), in which replicated bacteria draw up in a line along their long axes in close parallel arrangement, assuming the form of ropes), which were described previously [see (10, 11, 45, 47)], are presented on the graphs and in the tables below the graphs in **Figure 1A**. Recall that the *Mtb* belonged to the Beijing genotype family for most patients, excluding patients 3 and 4 (**Supplementary Table 1**).

Several types of macrophage subpopulations were determined in an immunofluorescence assay (**Figures 1A, B**). Alveolar macrophages from the lung tissues of patients 3 and 17 were characterized by a higher number of *Mtb*-infected cells (>1% of the total macrophages examined) and expressed pro-inflammatory or anti-inflammatory cytokines in the cytoplasm and microdomains of host and other non-infected cells. Although *Mtb* belonged to different genotype families and differed in phenotypic states between the patients, the pathogen could not resume replication in the prolonged *ex vivo* cell cultures after withdrawal of anti-TB drugs for both patients and, therefore, only survived in colonies, including those with cording morphology (patient 3). In the *ex vivo* cultures of macrophages obtained from the lung tissues of patients 1, 2, 4, 11–13, and 19–21, the number of cells with *Mtb*, which were characterized by different phenotypic and growth features among the patients, ranged from 0.11% (patient 12) to 0.68% (patient 11). A significant number of cells expressed both M1 and M2 cytokines in the same cell populations. For patients 16 and 18, the cytokine-expressed cells were rare in the *ex vivo* cell cultures. Despite the small number of the *Mtb*-infected cells detected for patient 18 (only 0.37%), the pathogen resumed active growth in the host cells in the prolonged *ex vivo* cell culture under antibiotic-free conditions. In these macrophage populations, *Mtb*, – whether solitary or as colonies, with or without cording morphology – were found in host cells expressing both anti-inflammatory/pro-bacterial M2 and pro-inflammatory/anti-bacterial M1 cytokines for all patients studied (**Figure 1B**).

According to our findings, the frequency of macrophages expressing M1 or M2 cytokines among the patients studied is likely to be irrespective of specific features of the pathogen in host cells, but may be determined by other factors, including those related to the local lung environment. On the other hand, specific variations in cytokine signaling could directly affect some properties of the pathogen that are crucial for controlling *Mtb* infection in the lungs of TB patients.

### 3.2 Smoker’s macrophages with activation of the AhR pathway are the main subset of cells in the macrophage populations from different lung TB lesions of the patients

**Figure 2:**
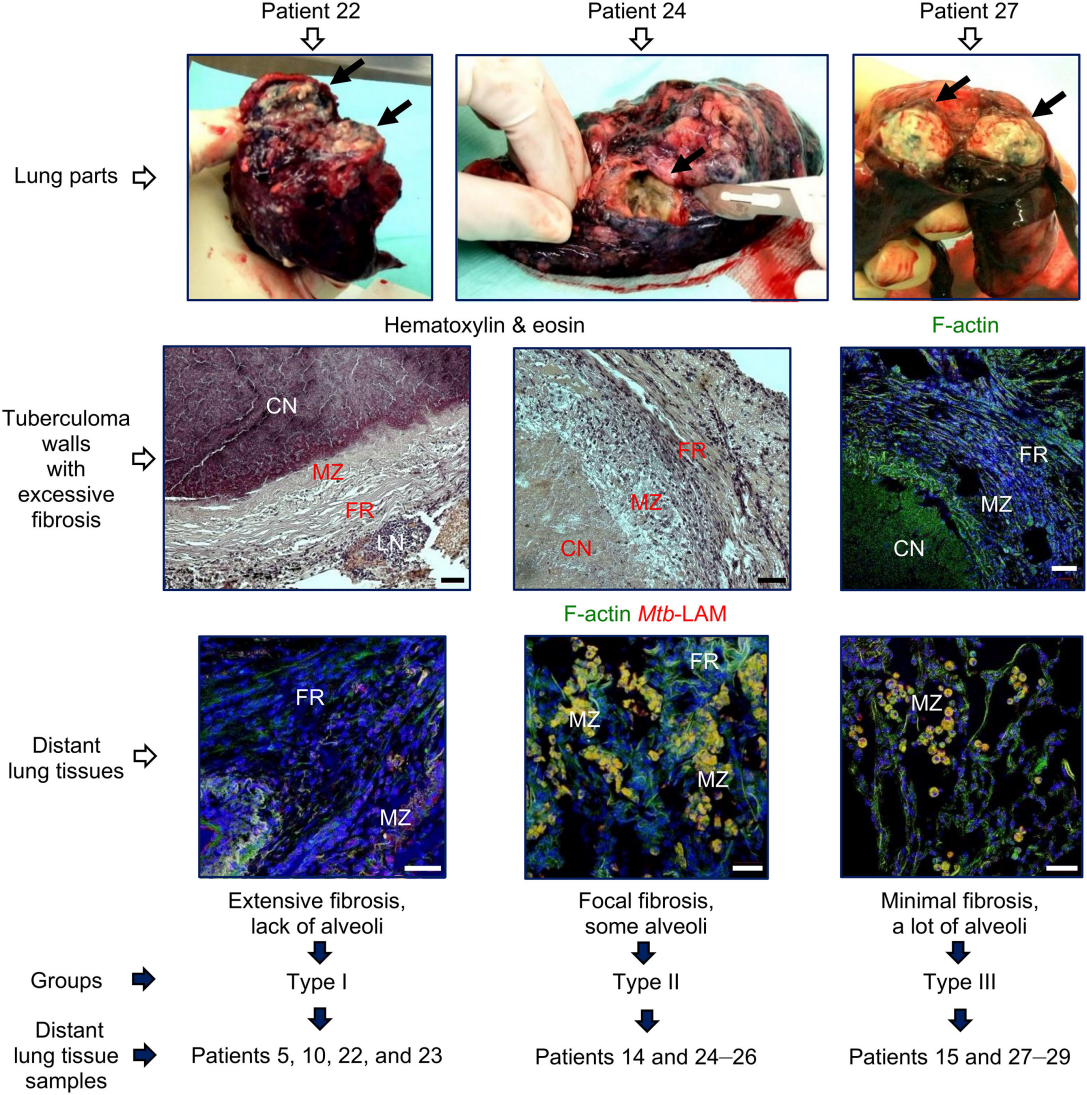
Histopathological examination of the tuberculoma wall and distant lung tissue samples obtained from the same lung parts surgically removed from TB patients demonstrate varying degrees of fibrosis in the discrete TB lesions. Representative light and confocal 2D merged fluorescent images of the histological sections stained with hematoxylin & eosin or by antibodies reacting with *Mtb*-LAM (red signal) and/or Alexa 488-conjugated phalloidin (green signal) are shown. Nuclei are stained by DAPI (blue signal). Tuberculomas are indicated by black arrows. CN, caseous necrosis; FR, fibrotic region; LN, lymphonodus; MZ, macrophage-rich zone. The scale bars are 50 μm each.

Within a single patient, pulmonary TB lesions range from the recruitment of multiple immune cells to the alveoli, abnormal repair of damaged lung parenchyma resulting in focal or extensive tissue fibrosis, aggregation of immune cells into small granulomas (size: 1–5 mm in diameter) to more differentiated tuberculomas (size: >12 mm in diameter) with a thicker dense wall in fibrous encapsulation and a large mass of central caseum, which may undergo liquefaction to form cavities filled with replicating *Mtb* that are released into nearby airways through coughing and infect healthy people through droplet dispersal (31–33, 48, 49). Therefore, to find out which pathomorphological factors and molecular events influence cytokine signaling during *Mtb* infection and active TB disease in the patients’ lungs, we examined macrophages from (1): distant lung tissues of patients 5, 10, 14, 15, 22–29, and, in parallel, (2) tuberculoma walls of patients 22–29. Since the histological and morphological examination of the distant lung tissue samples showed considerable variation in the extent of fibrosis among the patients, as described previously [see (44)], the specimens from patients 5, 10, 22, and 23 with extensive fibrosis and the absence of alveoli were assigned to the “Type I” group (**Figure 2**). The specimens from patients 14, 24–26 with focal fibrosis and some alveoli as well as patients 15 and 27–29 with minimal signs of fibrosis and numerous alveoli were assigned to the “Type II” and “Type III” groups, respectively (**Figure 2**).

**Figure 3:**
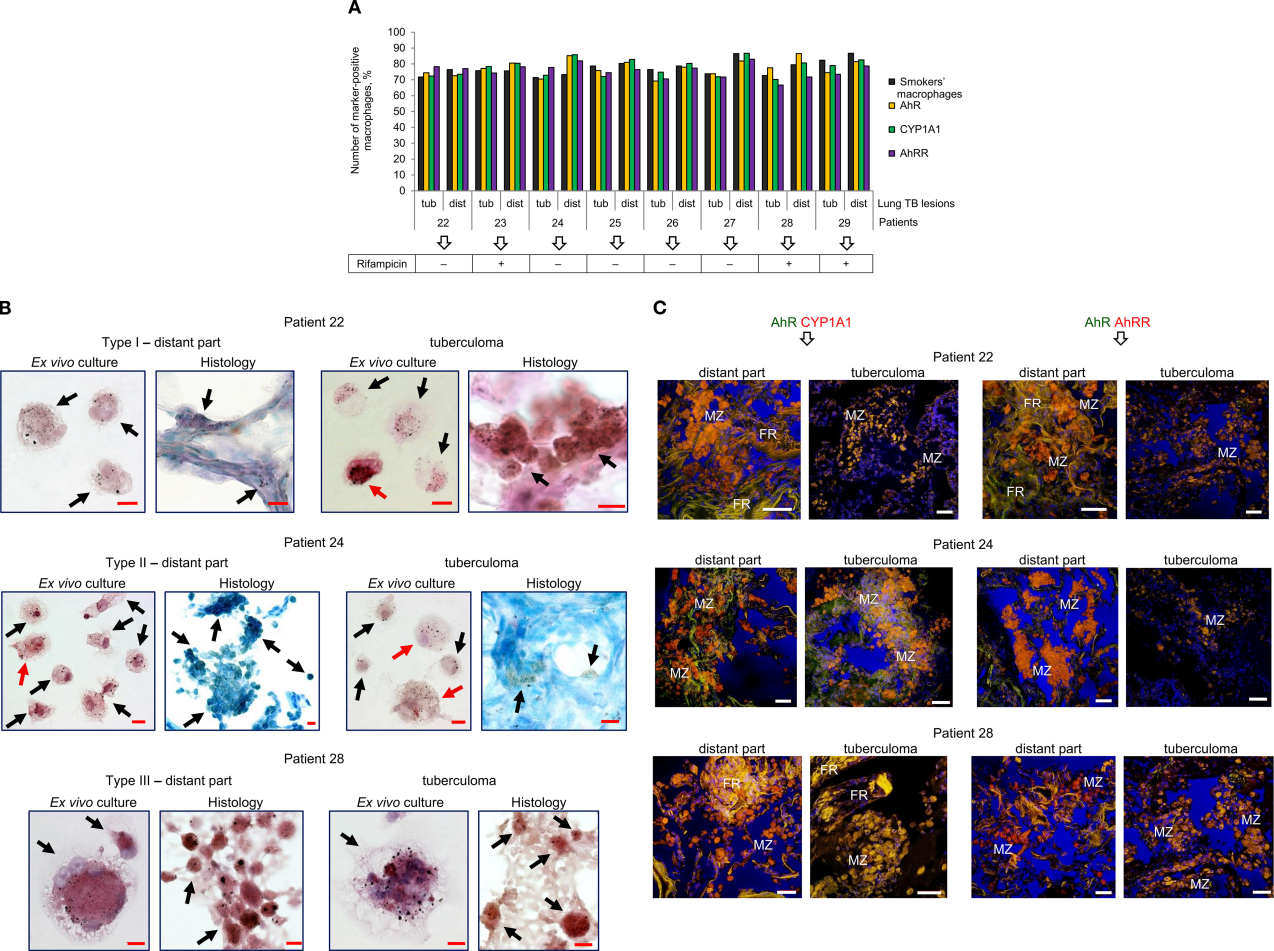
Smoker’s macrophages express the AhR pathway-specific markers in various lung TB lesions regardless of the presence or absence of rifampicin in an anti-microbial chemotherapy of the patients. **(A)** The number of smoker’s macrophages with denser dark inclusions in the cytoplasm and the marker-positive macrophages expressed as the percentage of the total number of the patients’ macrophages analyzed after *ex vivo* culture for 18 hours and on the histological sections obtained from the same tuberculoma wall (tub) and distant lung tissue (dist) samples, respectively. The table below a graph shows the use of rifampicin in the sets of anti-TB drugs for the patients’ treatment before surgery: (+), yes (–); no. **(B)** Representative images of smoker’s macrophages stained by the ZN method and analyzed after *ex vivo* culture for 18 hours and, in parallel, on the histological sections obtained from the same lung tissue samples are shown. Red and black arrows point to smoker’s macrophages (solitary or in clusters) with acid-fast *Mtb* (solitary or as colonies) and without the pathogen in them, respectively. Scale bars are 10 μm each. **(C)** Representative confocal merged fluorescent images of the macrophages stained by antibodies reacting with human AhR (green signal) and CYP1A1 or AhRR (red signal) demonstrate colocalization of these markers (yellow signal) on the histological sections of all lung TB lesions. Nuclei are stained by DAPI (blue signal). Collagen fibers are strongly autofluorescent (yellow signal) on histological immunofluorescent images. FR, fibrotic region; MZ, macrophage-rich zone with the marker-positive cells. Scale bars are 50 μm each.

In our study of the macrophage populations from different lung TB lesions of the patients, many macrophages with a large number of denser dark inclusions in the cytoplasm were identified and were what is called smoker’s macrophages, i.e., alveolar macrophages, with or without *Mtb* in them, as the main subset of cells not only in distant lung tissues from the “Type II” and “Type III” groups with multiple alveoli, but also in tuberculoma walls for patients 22–29 and distant lung tissues from a “Type I” group without alveoli for patients 22 and 23 both in the *ex vivo* cell cultures and, in parallel, on the histological sections (**Figures 3A, B**). Of note, some smoker’s macrophages were seen in the interstitial areas of the lung specimens of these patients, too (**Figure 3B**). Also, numerous smoker’s macrophages were observed both in the alveolar/interstitial areas and in small granuloma with a necrotic mass in the center on the histological preparations obtained from the distant lung tissue sample of patient 14 (**Supplementary Figure 2**).

Exogenous molecules, such as one of the main components of tobacco smoke and environmental carcinogen benzo[*a*]pyrene (50, 51), anti-TB drugs rifampicin and rifabutin (52), a redox cycling pigment phthiocol in the *Mtb* cell wall (53), bind to AhR, an evolutionarily conserved, ligand-activated transcription factor that regulates not only xenobiotic metabolism, but also plays important roles in the modulation of host immune response (54) and host-microbe interactions (55), including *Mtb* infection in macrophages (56). Upon ligand binding, AhR translocates from the cytoplasm into the nucleus and initiates the transcription of target genes, encoding detoxifying cytochrome P450 monooxygenase CYP1A1, its own negative regulator AhRR, and both pro-inflammatory cytokines IFNγ, TNFα, IL-1β, IL-12 and anti-inflammatory/immunosuppressive cytokine IL-10, among others (51–56). The AhR–AhRR–CYP1A1 axis also provides a potential target for therapeutic immunomodulation that should consider the ligand-, cell-type-, tissue microenvironment-specific, and species-dependent effects of the AhR-induced programs (51–57). In our study, a majority of macrophages expressed AhR, AhRR, and CYP1A1 in the cytoplasm of cells on the histological sections obtained from all lung tissue samples of patients 22–29 despite the different pathomorphological characteristics of the lung TB lesions examined and the presence or absence of rifampicin in the courses of intensive anti-TB chemotherapy of these patients (**Figures 3A–C**; **Supplementary Table 1**). The AhR-, AhRR-, and CYP1A1-expressed macrophages were identified not only in the alveolar/interstitial zones, but also in fibrotic area of small granuloma on the histological sections obtained from the lung tissue sample of patient 14 (**Supplementary Figure 2**). Of note, these immune regulation-related markers were not expressed in other immune cells (neutrophils and lymphocytes), as well as in fibroblasts and pulmonary epithelial cells.

Thus, smoker’s alveolar macrophages, localizing in various lung tissues with or without alveoli, demonstrate activation of the AhR pathway in all lung TB lesions of the patients studied.

### 3.3 The macrophage populations exhibit the lesion-specific cytokine profiles for the same patients

**Figure 4:**
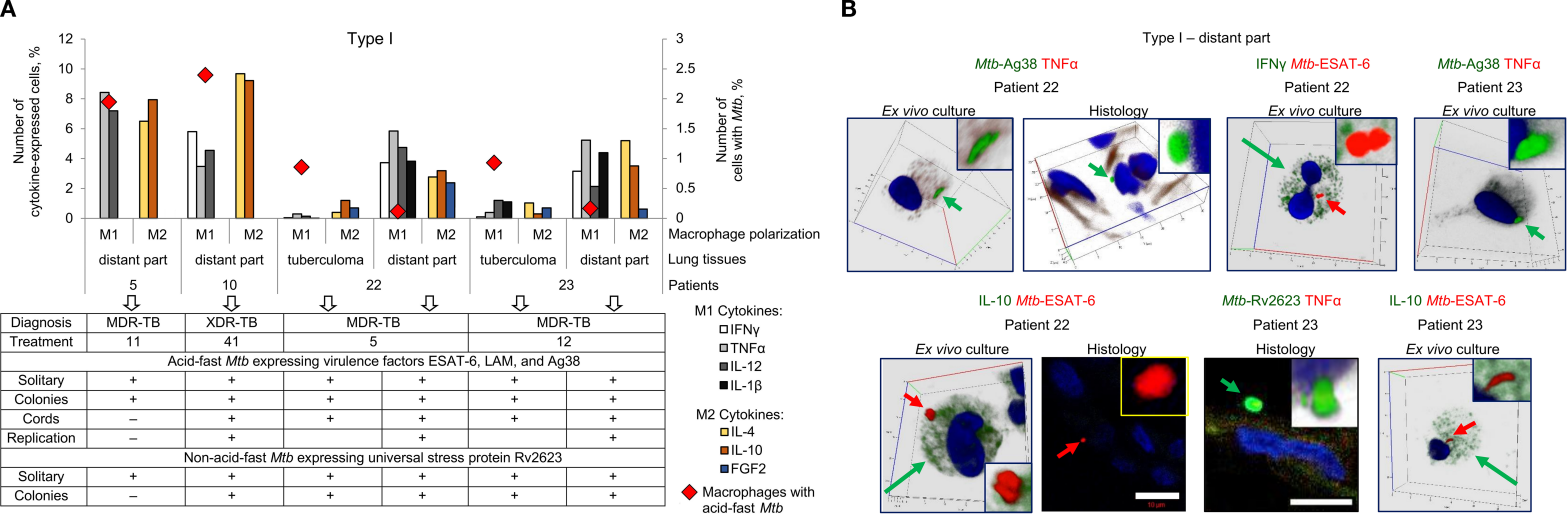
The cytokine-produced macrophages with or without *Mtb* in them are rare in the distant lung tissues of “Type I” group as well as in tuberculoma walls for the same patients. **(A)** The number of macrophages with the markers or acid-fast *Mtb* (solitary or as colonies) in them expressed as the percentage of the total number of the patients’ macrophages analyzed after *ex vivo* culture for 16–18 hours. The table below the graph shows some characteristics of the patients and pathogen at different lung TB lesions: (+), yes (–); no. Anti-TB treatment in the months. **(B)** Representative confocal 3D and 2D merged fluorescent images demonstrate the cells stained by antibodies reacting with human cytokines and *Mtb* antigens (green or red signals) and analyzed after *ex vivo* culture for 18 hours and, in parallel, on the histological sections obtained from the same lung tissues. Nuclei are stained by DAPI (blue signal). Short arrows (green or red) point to *Mtb*, solitary or as colonies. Enlarged images of *Mtb* are shown in the upper or lower right corners. Long green arrows point to the cytokine-positive macrophages with *Mtb* in them. The scale bars are 10 μm each.

**Figure 5:**
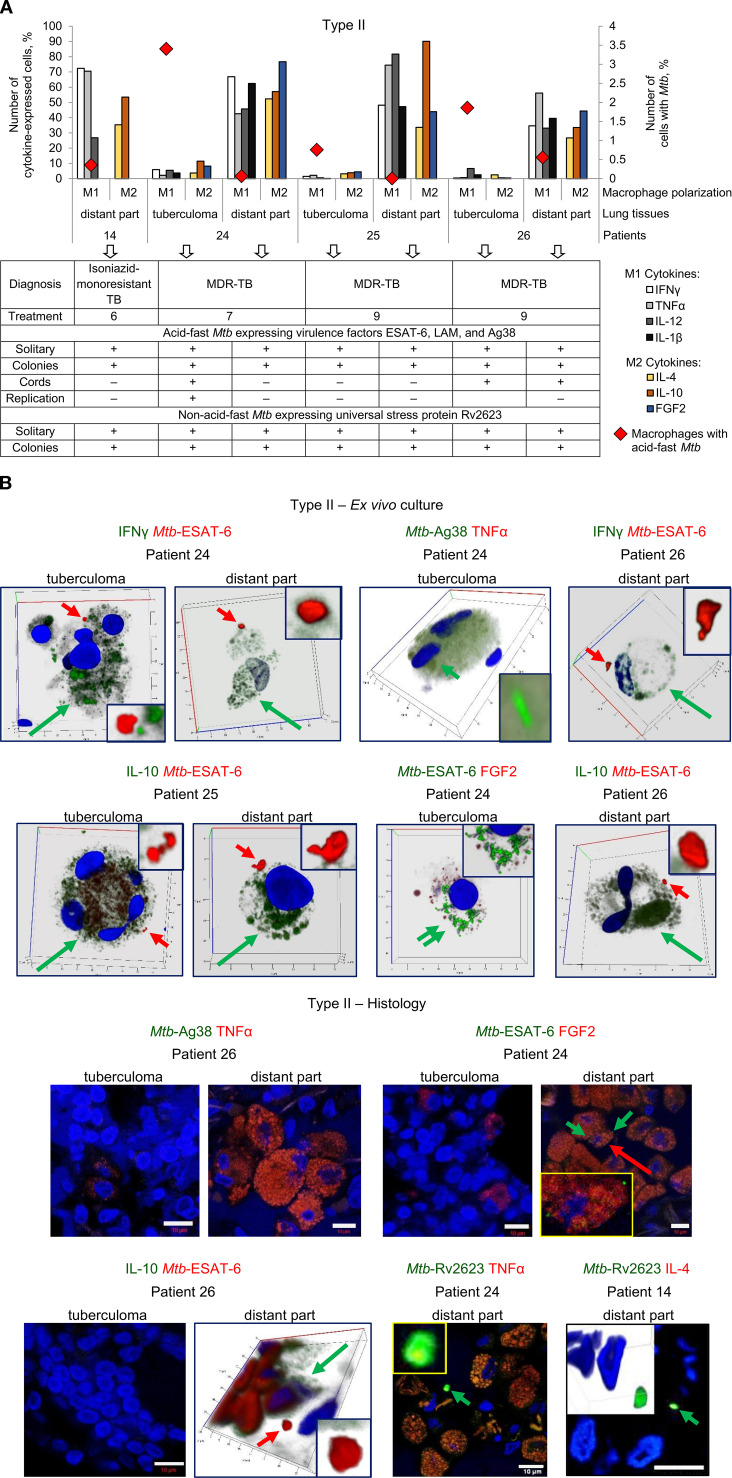
The *Mtb*-infected macrophages with production of M1 and M2 cytokines are identified predominantly in the distant lung tissues of “Type II” group, but not in tuberculoma walls for the same patients. **(A)** The number of macrophages with the markers or acid-fast *Mtb* (solitary or as colonies) in them expressed as the percentage of the total number of the patients’ macrophages analyzed after *ex vivo* culture for 18 hours. The table below the graph shows some characteristics of the patients and pathogen at different lung TB lesions: (+), yes (–);, no. Anti-TB treatment in the months. **(B)** Representative confocal 3D and 2D merged fluorescent images demonstrate the cells stained by antibodies reacting with human cytokines and *Mtb* antigens (green or red signals) and analyzed after *ex vivo* culture for 18 hours and, in parallel, on the histological sections obtained from the same lung tissues. Nuclei are stained by DAPI (blue signal). The abbreviation as in **Figure 4B**. The scale bars are 10 μm each.

**Figure 6:**
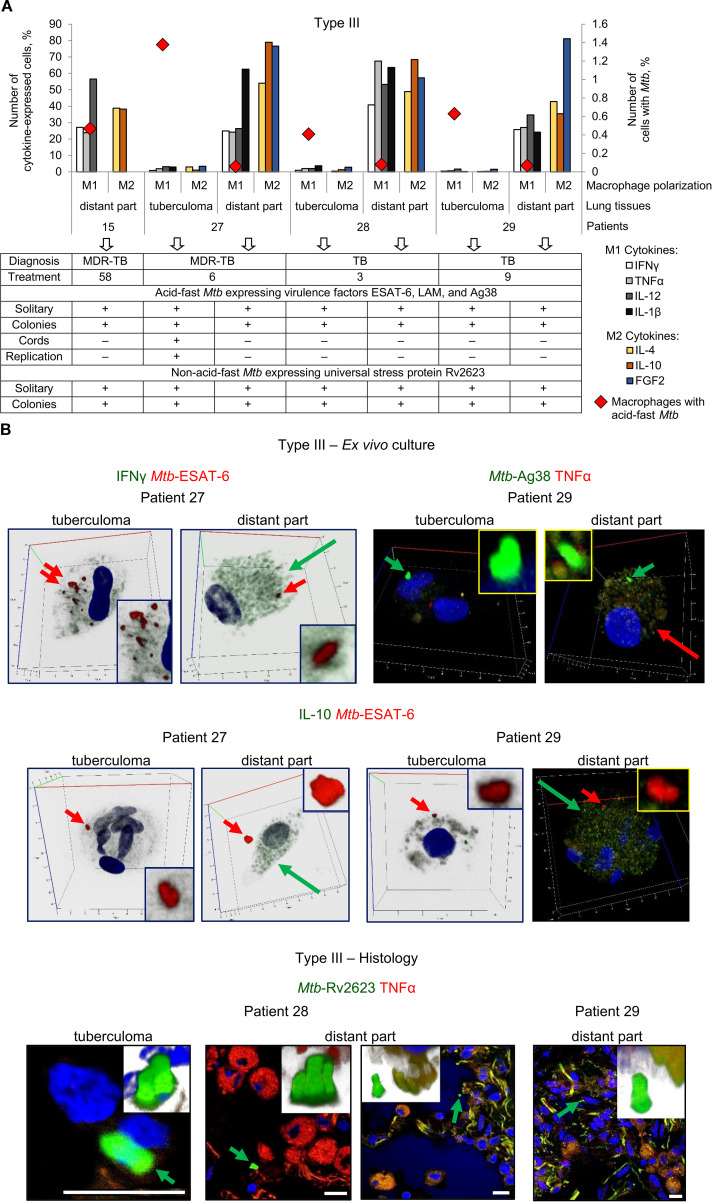
Alveolar macrophages with expression of M1 and M2 cytokines are also observed in the distant lung tissues of “Type III” group and are mainly absent in tuberculoma walls for the same patients. **(A)** The number of macrophages with the markers or acid-fast *Mtb* (solitary or as colonies) in them expressed as the percentage of the total number of the patients’ macrophages analyzed after *ex vivo* culture for 18 hours. The table below the graph shows some characteristics of the patients and pathogen at different lung TB lesions: (+), yes (–);, no. Anti-TB treatment in the months. **(B)** Representative confocal 3D and 2D merged fluorescent images demonstrate the cells stained by antibodies reacting with human cytokines and *Mtb* antigens (green or red signals) and analyzed after *ex vivo* culture for 18 hours and, in parallel, on the histological sections obtained from the same lung tissues. Nuclei are stained by DAPI (blue signal). Collagen fibers are strongly autofluorescent (yellow signal) on histological immunofluorescent images. The abbreviation as in **Figure 4B**. The scale bars are 10 μm each.

In the macrophage populations from the lung tissue samples of tuberculoma walls and distant parts from the “Type I”, “Type II”, and “Type III” groups, the cytokine profiles and *Mtb* burden were also examined in an immunofluorescence assay with simultaneous staining of one of the *Mtb* markers (ESAT-6, LAM, Ag38, and Rv2623) and one of the M1 (IFNγ, TNFα, IL-12, and IL-1β) or M2 (IL-4, IL-10, and FGF2) cytokines in various combinations both on the cytological preparations after *ex vivo* culture for 16–18 hours and, in parallel, on the histological sections for each patient. However, in this work, the Rv2623-positive *Mtb* in host cells were analyzed only on the histological preparations. The exact number of macrophages expressing cytokines, which were accumulated in the cytoplasm and/or numerous discrete granules, was evaluated in the *ex vivo* cell cultures (**Figures 4A**, **5A**, **6A**), since the data obtained in the analysis of the cytological and, in parallel, histological preparations were generally the same for each patient (**Figures 4B**, **5B**, **6B**). Importantly, no autofluorescent signals were detected on denser dark inclusions in the cytoplasm of macrophages for both these patients and the patients studied above (**Supplementary Figure 3**).

While the pathogen belonged to the same Beijing genotype family and exhibited similar phenotypic and growth features (**Supplementary Table 1**), as well as approximately the same spectrum of drug-resistance mutations in *Mtb* genes [see Table 1 in (45)], in the lungs of the patients studied, the cytokine expression patterns differed significantly between the identified pathomorphological groups (**Figures 4A**, **5A**, **6A**). Very few macrophages (<10% of the total macrophages examined) expressed both M1 and M2 cytokines in the “Type I” cell populations as well as from tuberculoma walls for the same patients (**Figures 4A, B**). The strongest cytokine signaling, with the macrophages producing both M1 and M2 cytokines (30-90% of cytokine-positive cells for each cytokine examined), and low *Mtb* burden were detected in all “Type II” and “Type III” cell populations, but not in tuberculoma walls, which had very small numbers of macrophages with any cytokine expression and significantly higher *Mtb* loads in them for the same patients (**Figures 5A, B**, **6A, B**). The pathogen – whether solitary and in colonies, with or without cording morphology, with the expression of the virulence factors ESAT-6, LAM, Ag38 or the universal stress protein Rv2623 – was revealed in both M1 and M2 cytokine-positive macrophages as well as in cytokine-negative host cells in both cytological and histological preparations for the same patients (**Figures 4B**, **5B**, **6B**).

Thus, our data indicate a noticeable heterogeneity of cytokine signaling activity in macrophages between the lung TB microenvironments with different pathomorphological characteristics, thereby demonstrating distinct patterns of immune reactions that are believed to reflect the complex interactions of the patient’s tissues with the pathogen and may affect disease control or exacerbation at specific sites of *Mtb* infection.

### 3.4 Alveolar macrophages co-express the M1- and M2-associated cytokines through simultaneous production in the same cells of TB patients

**Figure 7:**
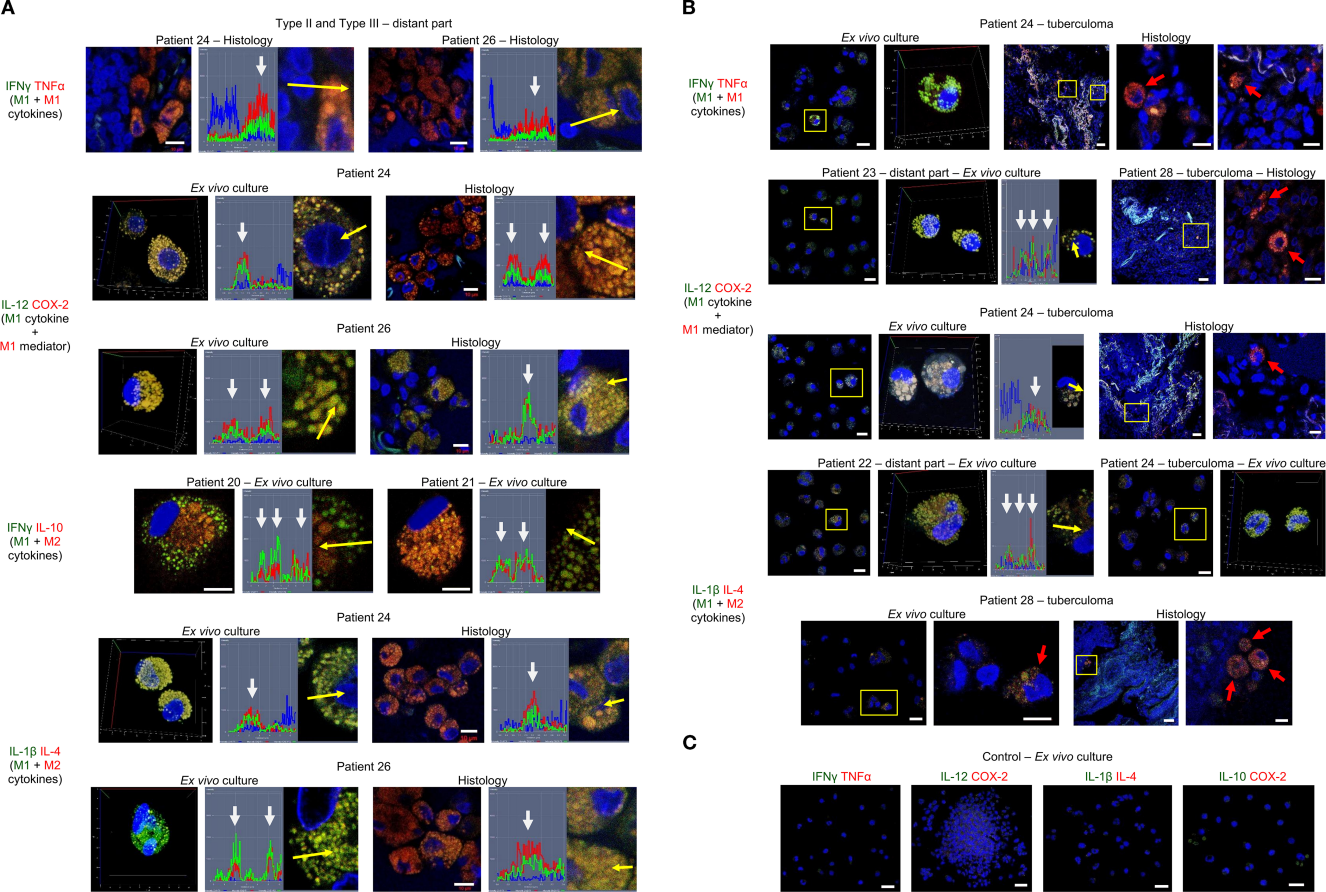
**(A, B)** Alveolar macrophages of TB patients co-express different M1 and M2 cytokines as well as various M1 cytokines and mediators in the activated cells both in the distant lung tissues and in tuberculoma walls, but not **(C)** in the monocytes and neutrophils of a control group. **(A–C)** Representative confocal 3D and 2D merged fluorescent images of the cells stained by antibodies reacting with human cytokines and COX-2 (green or red signals) and analyzed after *ex vivo* culture for 18 hours and, in parallel, on the histological sections obtained from the same tissue samples demonstrate colocalization of the analyzed markers (yellow signal). Nuclei are stained by DAPI (blue signal). **(A, B)** To the right of the immunofluorescent images: profile images of some macrophages are shown. Yellow arrows point to the areas for constructing profile graphs. White arrows point to the colocalization of the markers in the cytoplasm and vesicles of cells in the graphs of profile images. **(B)** Close-ups of the parts of the images with alveolar macrophages co-expressing the examined markers on the left panel are shown in the central and/or right panels. Some alveolar macrophages with co-expression of the cytokines are indicated by red arrows. The scale bars are **(A)** 10 μm each, **(B, C)** 20 μm and 50 μm each on the general images taken from the cytological and histological preparations, respectively, and **(B)** 10 μm each on the parts of the images shown in the central and/or right panels.

As the distant lung tissues from the “Type II” and “Type III” groups as well as from patients 1–4, 13, 20, and 21 were characterized by >60% of macrophages expressing both M1 and M2 cytokines in the same cell populations (**Figures 1A**, **5A**, **6A**), the co-expression of the cytokines of both types in cells was estimated in an immunofluorescence assay with simultaneous staining of one of the M1 cytokines (IFNγ, TNFα, IL-12, and IL-1β) and one of the M2 cytokines (IL-4 and IL-10) or with simultaneous staining of two of the M1 cytokines (IFNγ, TNFα, and IL-12) or mediators (COX-2) in various combinations both on the cytological preparations after *ex vivo* culture for 16–18 hours for all patients and, in parallel, on the histological sections obtained from the specimens assigned to various pathomorphological groups (**Figures 7A, B**). The leukocytes from blood samples of healthy volunteers were taken as a control group and analyzed in parallel after *ex vivo* culture for 18 hours and the identical dual-staining of cytokines/mediators in the same combinations as for the patients’ macrophages (**Figure 7C**).

To our surprise, the co-expression of not only different M1 cytokines/mediators, but also M1 and M2 cytokines was detected in macrophages on both the cytological and, in parallel, histological preparations obtained from the same distant lung tissues from the “Type II” and “Type III” groups and for other patients studied (**Figure 7A**). In contrast to these findings, the vast majority of macrophages from tuberculoma walls and a “Type I” group of TB patients (**Figure 7B**), as well as all monocytes and neutrophils obtained from blood samples in a control group (**Figure 7C**) did not produce either M1 or M2 cytokines in any of the preparations examined. However, rare cytokine-positive macrophages from these samples also co-expressed M1 and M2 cytokines and were found among cytokine-negative cells on all preparations examined (**Figure 7B**). Consequently, this finding indicates the phenomenon of simultaneous production of M1 and M2 cytokines by the same macrophages and, with high probability, excludes the occurrence of the M1/M2-positive cells merely as a result of cytokine uptake *via* endocytosis, if cytokines of both types were synthesized by differently activated cells in the patients’ lung tissues.

Altogether, our data indicate that the macrophages, co-expressing M1 and M2 cytokines and showing a mixed M1/M2 polarization state, are activated in the lungs of TB patients before surgery, but not as a result of cell processing in *ex vivo* culture. With high probability, the M1 and M2 cytokines are simultaneously produced by the same macrophages in the lungs of the patients studied. Overall, identification of the mixed M1/M2-polarized alveolar macrophages, as well as cells without any cytokine expression (which share an M0-like polarization state), demonstrates the complex and dynamic features of the immune response in local lung environments during human TB disease.

### 3.5 Differential regulation of the cytokine and NF-*κ*B signaling activities in macrophages affects the control of *Mtb* infection in various lung TB lesions of the patients

**Figure 8:**
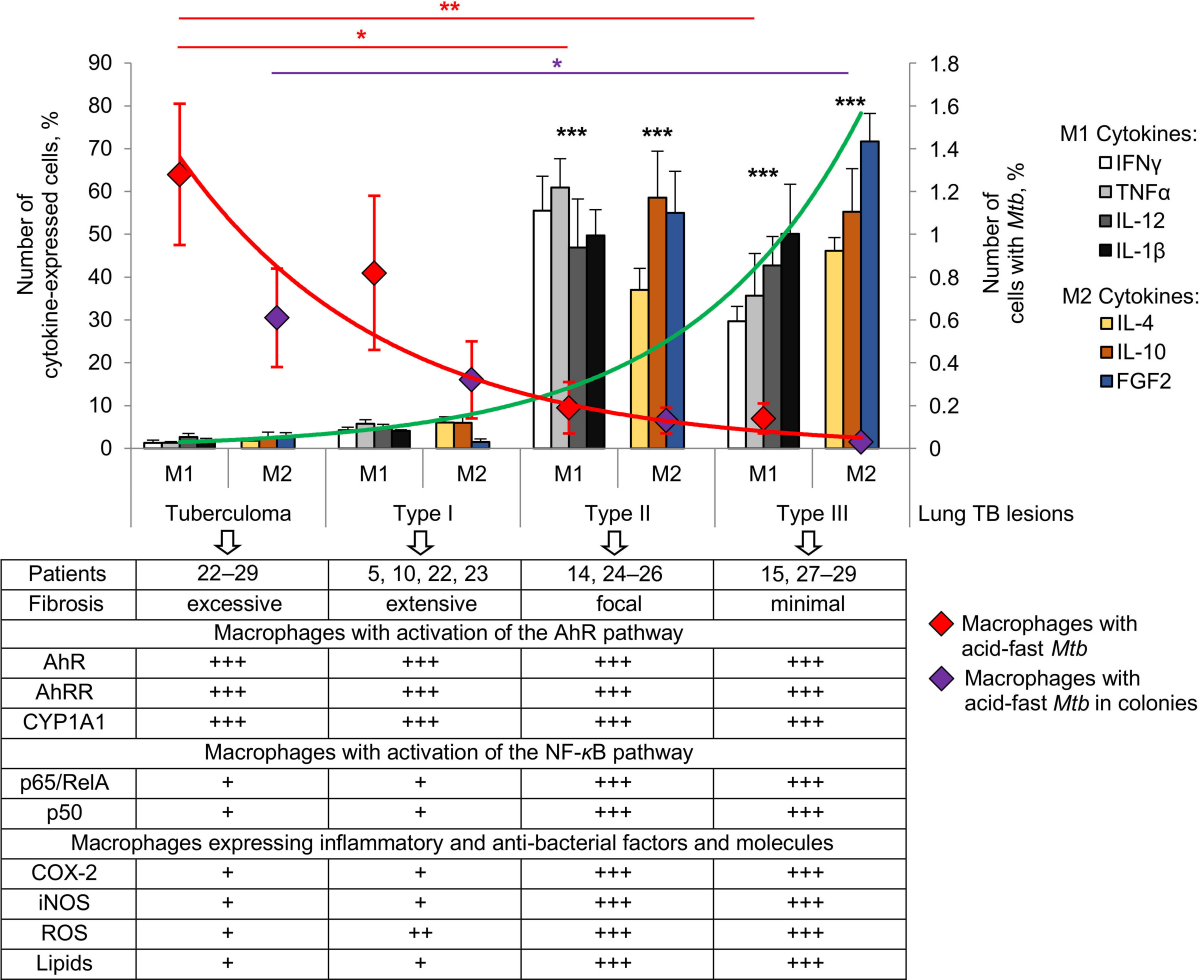
The NF-*κ*B-activated macrophages with a mixed M1/M2 polarization state from the “Type II” and “Type III” groups of the lung TB lesions control *Mtb* infection in contrast to bactericidal-inactive cells in an M0-like polarization state from the “Type I” and “Tuberculoma” groups for the same patients. The number of macrophages with any *Mtb* (single or as colonies) and with the colonies of *Mtb*, both expressed as the percentage of the total number of alveolar macrophages. The number of cytokine-positive cells expressed as the percentage of the total number of the patients’ macrophages analyzed. Data are expressed as the means ± SEM. Below the graph, the table presents the characteristics of the lung tissues and macrophages in the groups of the patients’ lung TB lesions, including the extent of fibrosis and activation of signaling and regulatory pathways, inflammatory and anti-bacterial factors. Symbols (+), (++), and (+++) indicate activation of these factors (a semi-quantitative analysis) in approximately 0.5-5%, 10-40%, and 50-80% of cells, respectively. Red and green curve lines indicate the trends in the alteration of the *Mtb* burden and cytokine profiles, respectively, in macrophages between various groups of the lung TB lesions. For *Mtb* loads (comparisons in the groups – red lines for the number of *Mtb*-infected macrophages and blue line for the number of macrophages with the colonies of *Mtb*): *P < 0.05, **P < 0.01, Student’s *t*-test. For cytokine profiles (comparisons of the number of the cytokine-positive macrophages in “Tuberculoma” or “Type I” and “Type II” or “Type III” groups): ***P < 0.001, Student’s *t*-test.

To estimate the influence of cytokine signaling and activation of regulatory pathways on bacterial loads at the sites of TB infection with different pathomorphological features, a new “Tuberculoma” group, consisting of lung specimens obtained from the tuberculoma walls of patients 22–29 (*n* = 8) and characterized by excessive fibrosis, was added to the “Type I”, “Type II”, and “Type III” groups (**Figure 8**). The detailed pathomorphological characteristics of the lung tissues from “Tuberculoma” group were described in **Figure 2**.

During *Mtb* infection in animal lungs, activation of the master pro-inflammatory transcriptional regulator NF-*κ*B results in the release of many pro-inflammatory factors and molecules, including the enzymes COX-2 and iNOS, which are responsible for the production of prostaglandin E2 and microbicidal nitric oxide (NO), respectively, and cytokines, including TNFα, IL-1β, and IL-12, in the lung macrophages (4, 16, 22, 58). Prostaglandin production through the use of lipids and generation of NO and ROS are believed to play a central role in the anti-microbial activation of macrophages and elimination of intracellular pathogens (4, 8, 58). At the same time, simultaneous activation of the AhR and NF-*κ*B pathways may lead to synergistic or antagonistic effects on specific target genes, changing their expression in cells (59). Therefore, data on activation of NF-*κ*B p65/RelA and p50 subunits, COX-2, iNOS, ROS, and lipids in the macrophages from the examined groups, assessed in our previous works [see (11, 44, 45)], and AhR signaling are presented in the table below the graph in **Figure 8**.

The lack of production of inflammatory and anti-bacterial factors and the elevated *Mtb* loads (*P < 0.05 and **P < 0.01, **Figure 8**), including the pathogen in colonies (*P < 0.05, **Figure 8**), in the macrophages from the “Tuberculoma” and “Type I” groups characterized by highly fibrotic lung tissues correlated with the absence of both M1 and M2 cytokine-expressed and NF-*κ*B-produced cells. Conversely, the macrophages with simultaneous high production of M1 and M2 cytokines (***P < 0.001, **Figure 8**), NF-*κ*B subunits, and abundant pro-inflammatory and bactericidal molecules in the same cells showed the lowest level of *Mtb* infection in the “Type II” and “Type III” groups with the lowest degrees of fibrosis in the lung tissues. No changes in the activation of the AhR pathway were found in macrophages for all groups examined (**Figure 8**).

Thus, the *Mtb* burden is observed in the patients’ macrophages showing an M0-like polarization state without production of both pro-inflammatory/anti-bacterial M1 and anti-inflammatory/pro-bacterial M2 cytokines, probably as a result of inhibiting NF-*κ*B-mediated processes, in tuberculoma walls and extensively fibrotic tissues with the absence of alveoli. For the same patients, smoker’s macrophages co-expressing M1 and M2 cytokines and NF-*κ*B-related molecules and exhibiting a mixed M1/M2 polarization state can control, but not completely eliminate, *Mtb* infection in some lung TB lesions with minimal fibrosis and multiple alveoli. With high probability, the expression of the AhR pathway-specific markers in a majority of macrophages from all lung TB lesions examined does not affect the cytokine signaling regulation in the lungs of the patients studied. Overall, the inability of host cells to clear *Mtb* indicates a generally non-optimal development of the immune responses to the pathogen at various foci of TB disease in the patients’ lungs.

## 4 Discussion

Nowadays, TB is an infectious disease that, despite the availability of antibiotic therapy, seriously threatens global population health and human life (1–3). The emergence of drug-resistant *Mtb* strains has posed new challenges to TB prevention and control for achieving the WHO’s goal of having TB eradicated by 2035 (1–3). Both *in vitro* and *in vivo* studies using immune cell cultures and animal models, including gene knockout mice and non-human primates, have contributed to the current understanding of host-pathogen interactions in TB and have demonstrated that cytokines play an important role as immune-regulatory factors in controlling *Mtb* infection and restoring tissue homeostasis after pathogen clearance (18–22, 60, 61). However, none of these models fully recapitulate the pathological spectrum as well as the phenotypic and growth features of the pathogen in clinical TB cases, observed in patients who have often experienced long-lasting *Mtb* infection and have undergone several rounds of months-long antibiotic therapy (60, 61). The limited understanding of the cytokine landscape and mechanisms of its regulation underlying the local host-pathogen dynamics within specific foci of pulmonary TB hampers the development of HDT, especially cytokine therapy for modulating the immune system of TB patients, and its application in human treatment (26–29, 62).

In this work, we have for the first time evaluated cytokine signaling activation in the control of *Mtb* infection in the lung macrophages, which were characterized as smoker’s alveolar macrophages in various lung TB lesions of patients with predominantly drug-resistant TB. According to our findings, specific features of *Mtb* in host cells did not affect the expression of both M1 and M2 cytokines by the patients’ macrophages. At the same time, we indicated polarized cytokine expression by macrophages from different sites of TB disease in the lungs of the same patients, when a majority of them from distant lung tissues with local/minimal fibrosis and preservation of alveoli co-expressed M1 (IFNγ, TNFα, IL-1β, and IL-12) and M2 (IL-4, IL-10, and FGF2) cytokines and could control *Mtb* infection, but did not completely eradicate the pathogen. In the same lungs, smoker’s macrophages from the tuberculoma walls and distant lung tissues with excessive/extensive fibrosis and without alveoli did not produce both M1 and M2 cytokines and were, especially in tuberculomas, the main niche for *Mtb* survival and replication in host cells among various lung tissue environments examined. These features of macrophage polarization were associated with the presence or absence of the NF-*κ*B-dependent activation of macrophages, but not with AhR-related signaling activities that were determined for a majority of the patients’ macrophages in all lung TB lesions examined. These results are very important for understanding the role of the NF-*κ*B and AhR signaling pathways in the control of *Mtb* infection during human TB, because the modulation of their activity has shown various effects: promote or control, often opposite, – on mycobacterial infection in different cell lines and rodent and fish model systems (52, 53, 56–58, 63–66).

For TB patients studied in our work, the pro-inflammatory- and microbicidal-activated macrophages co-expressed M1 and M2 cytokines with opposing activities simultaneously in the same cells, although only M1-associated cytokine expression was expected in these cells. In other studies of human TB, Pavlova and the co-workers (67) determined, with qPCR, high expression levels of the *TNFa* and *TGFb1* genes (for M1 cytokine TNFα and M2 marker transforming growth factor β (TGFβ), respectively) in the same samples that, according to their pathomorphological characteristics, corresponded to the “Type II” and “Type III” lung tissues in our work. Using immunohistological technique, Hwang and the co-workers (68) quantified the expression of M2 marker CD163 in alveolar macrophages from the lung regions with TB pneumonia pathology, which were similar to “Type III” samples in our work. Notably, using *in situ* hybridization for TGFβ and IFNγ transcripts on histological sections of solid small granulomas, McCaffrey and the co-workers (40) found reduced M1 cytokine IFNγ and elevated M2 marker TGFβ production in cells. In these studies, the *Mtb* loads were not examined.

As far as other human disorders are concerned, we have previously revealed [see (69)] the co-expression of M1 and M2 cytokines and activation of AhR signaling in the tumor-associated macrophages from the patients’ samples of non-small cell lung cancer (NSCLC) – both on the *ex vivo* cell preparations obtained immediately after isolation of cancer and immune cells – and, in parallel, on histological sections of the same samples, where the tumor-associated macrophages were localized far apart among numerous cytokine- and AhR signaling-negative cancer cells, especially in squamous cell carcinoma samples. Macrophages with a mixed M1/M2 polarization state were detected in multiple sclerosis lesions (70) and dermal inflammatory infiltrate of psoriatic plaques (71), though these studies analyzed only M1 and M2 CD markers on histological preparations. Interestingly, mass cytometry analysis of CD markers identified two macrophage subsets with M1 and M2 phenotypes in atherosclerotic lesions (72). Subsequent single-cell RNA sequencing (scRNA-seq) of these macrophages revealed both pro-inflammatory and anti-inflammatory transcriptional signatures in the same cells, indicating their mixed M1/M2 polarization state (72). Similarly, scRNA-seq analysis of zebrafish granulomas induced by *M. marinum* infection detected a mixed expression of the genes for M1 cytokines (IFNγ, IL-1β, IL-12) and M2 cytokines (IL-4, IL-13) (73). Using scRNA-seq analysis of bronchoalveolar lavage cells, Dallmann-Sauer and the co-workers (74) observed a stronger IFN**γ** signature in alveolar macrophages after *ex vivo Mtb* challenge in persons with HIV/latent TB infection compared to those with HIV alone. Thus, macrophages exhibiting a mixed M1/M2 polarization state with co-expression of both markers/cytokines in the same cells have been identified in diverse pathological situations, including human pulmonary TB and animal TB models.

Of note, in our earlier work with NSCLC patients (69), we found smoker’s macrophages with the co-expression of M1 and M2 cytokines and activation of AhR signaling on the *ex vivo* cell preparations and, in parallel, histological sections from the lung tissue samples obtained from the lung part about 5 cm away from the tumors for the currently smoking patients, but not for patients that were former and never smokers, those alveolar macrophages were characterized by an M0-like polarization state [see **Figures 4**, **6A, B**, **Supplementary Figure 3A, B** in (69)]. However, Mitsi and the co-workers (25), by contrast, observed alveolar macrophages with combined M1 and M2 features (according to expression of different CD markers) in bronchoalveolar lavage from the lungs of healthy and non-smoking individuals. Nevertheless, Bazzan and the co-workers (75) also determined that in healthy lungs of non-smoking humans, most alveolar macrophages were iNOS- and CD206-negative cells, therefore, were neither M1 nor M2, respectively. With smoking and chronic obstructive pulmonary disease severity, a number of alveolar macrophages with M1 and M2 polarization states increased significantly and these cells expressed simultaneously M1 and M2 markers in the same macrophages. Perhaps, a reduced risk of developing a cytokine storm and severe pneumonia as well as disease-related death among current smoking patients with coronavirus disease 2019 (76, 77) could be related to activation of not only pro-inflammatory M1 molecules, but also anti-inflammatory/immunosuppressive M2 mediators, likely as a result of AhR signaling activity, in alveolar macrophages of these patients before coronavirus infection. In this regard, the phenomenon of smoker’s alveolar macrophages with a mixed M1/M2 polarization state should be considered within the concept of innate immune memory and trained immunity, when the long-term functional reprogramming of innate immune cells (primarily macrophages) following exposure to certain endogenous or exogenous stimuli, such as microbes and microbial-derived ligands, vaccines, and IFNγ, among others (78–81), can either enhance protection against repeated homologous or heterologous attack or suppress the immune response and impair pathogen clearance (78–81).

Therefore, it is extremely important to determine the reasons why alveolar macrophages activated for the production of numerous pro-inflammatory and microbicidal molecules and cytokines were unable to eradicate intracellular *Mtb* in the lungs of TB patients. Using scRNA-seq analysis, Piccolo and the co-workers (82) revealed inhibitory effects on opposing activation programs, with strong suppression of many genes and enhancers at the transcriptional and epigenomic levels in mouse bone marrow macrophages treated with a combination of M1 (IFNγ) and M2 (IL-4) cytokines. However, these effects did not abolish the activation of M1 and M2 programs, which co-existed and exhibited an overall dominance of the IFNγ-induced program over the IL-4-induced program in co-stimulated cells (82). In our work, we also observed strong activation of pro-inflammatory/microbicidal M1 program in M1/M2-polarized smoker’s macrophages that controlled, but did not completely eliminate, *Mtb* infection in TB patients’ lungs. We hypothesize that the same cellular regulatory mechanisms governing mixed M1/M2 cytokine-induced activation of signaling pathways under antagonistic signals [as identified in (82)], along, probably, with mechanisms involving microRNA and other epigenetic regulators of macrophage gene activity (83–85) and the effects of trained immunization (78, 79), may mediate crosstalk between opposing cytokine programs in TB patients’ cells. The precise mechanisms remain to be elucidated. For our study, scRNA-seq could definitively resolve whether alveolar macrophages (particularly from the “Type II” and “Type III” groups) simultaneously produce M1 and M2 cytokines or engulf them *via* endocytosis if secreted by distinct macrophage subsets producing either M1 or M2 cytokines. However, this requires future investigation.

As demonstrated in mouse models of TB (86, 87), TNFα expression is necessary for granuloma formation following *Mtb* infection, whereas a lack of this cytokine results in poorly formed granulomas with extensive necrotic regions containing high *Mtb* loads (88). In TB patients, we observed no TNFα expression by macrophages in tuberculoma walls, which harbored significantly higher *Mtb* loads (excluding necrotic centers). In other lung lesions from the same patients, TNFα and other M1-associated cytokine expression correlated with activation of pro-inflammatory/microbicidal pathways and *Mtb* control. With immunohistological analysis, Marakalala and the co-workers (89) also identified heterogeneous TNFα expression across human TB lesions, with macrophages at granuloma peripheries exhibiting a more anti-inflammatory proteomic signature. Thus, TNFα expression varies markedly between lung TB lesions, reflecting more complex and lesion-specific regulatory processes in TB patients than in animal TB models.

In our work, the cytokine expression profiles of the patients’ macrophages exhibiting the M0-like or mixed M1/M2 polarization state did not depend on the genetic or phenotypic properties of the pathogen infecting the patients’ lungs, although ESAT-6 expression by *Mtb* was associated with attenuation of the innate immune response in mouse cell cultures (90) and other immunomodulatory effects promoting pathogen survival within host cells (91). In addition, drug-resistant *Mtb* strains are considered to affect various aspects of the host-pathogen interactions and drive immune suppression in both the animal models of TB infection and human MDR-TB (7, 92). However, we have associated specific macrophage phenotypes only with the local microenvironments that were characterized by the distinct patterns of tissue fibrosis varying across various lung TB lesions of the same patients. While the pro-inflammatory-activated macrophages released IL-4, which provided protection against excessive inflammation and restoration of the normal tissue architecture (13–18), and FGF2, which promoted tissue repair through activation of fibroblast migration, proliferation, and overproduction of extracellular matrix components (93–96), the detection of mainly defective reparative processes with a wide range of fibrosis severity and alveolar destruction also indicates suboptimal development of the immune response in the lungs of the patients studied.

Unlike other studies of small granulomas that were extremely heterogeneous for some parameters even within a single host (39–42), our research has revealed fundamental immunological, functional, and pathobiological features of macrophage polarization, where smoker’s alveolar macrophages with a mixed M1/M2 polarization state and pro-inflammatory/microbicidal activation controlled *Mtb* infection in the alveolar tissues with focal/minimal fibrotic processes. For the same patients, lung samples with excessive/extensive fibrosis, including tuberculoma walls, showed cell deactivation/suppression, with smoker’s macrophages in a resting, non-polarized M0 phenotype lacking cytokine production but expressing CD14 (a marker common to all macrophage types), as reported previously [see (44)], and the Ahr signaling pathway molecules. Interestingly, Ramirez and the co-workers (97) identified a consistent upregulation of the genes, encoding AhRR and CYP1A1, across 30 tissues of tobacco smoking individuals. In our work, despite variation in *Mtb* loads across lesions, consistent trends have been observed: tuberculoma walls, with their *Mtb*-harboring smoker’s macrophages that have high mobility and can migrate between alveoli and other lung tissues, including the lung tumors (for example, in our earlier work [see (69)], the tumor-associated macrophages with acid-fast *Mtb* in them were found in the resected tumor sample of NSCLC patient with concurrent squamous cell carcinoma and active pulmonary TB [see in (**Supplementary Figure 4**)], exhibited both the highest bacterial burden and lowest cytokine activation. In humans, M0 macrophages are expected to maintain tissue homeostasis (13–17). However, their presence in the patients’ tuberculomas signals immune suppression in these TB lesions. This immune failure drives disease severity and transmission risk, particularly when tuberculomas progress to cavities containing maximal *Mtb* loads, enabling aerosol spread (18, 45, 47). The mechanisms—whether tobacco smoke exposure- and fibrosis-related (48, 96–99) or independent—and key signaling pathways that reprogram macrophages from a mixed M1/M2 polarization state to an M0-like polarization state with inhibiting NF-*κ*B-mediated processes, but not benzo[*a*]pyrene-induced AhR signaling-dependent stimulation, remain unknown. Future research should target these pathways to improve therapeutic approaches, TB outcomes and reduce the risk of complications.

Of note, besides the innate immune response (nonspecific/natural), the adaptive immune response (specific/acquired) plays an essential role in maintaining immune balance against *Mtb* infection in human lungs (4–6). In this work, we focused on analyzing the cytokine signaling exclusively in the patients’ macrophages, as these cells are the indispensable key players in both immune response types (4–6) and the primary cell type targeted by *Mtb* infection and responsible to pathogen uptake (18, 19, 44–47). The contribution of other immune cell types to TB immunity will be evaluated in future research.

The limitation of our study is the small number of lung tissue samples analyzed for each group. However, the same lesion-specific features of the cytokine signaling, NF-*κ*B-related activities and activation of AhR–AhRR–CYP1A1 axis were found in smoker’s macrophages of patients 32 and 33 with pulmonary TB, who had not been given anti-TB treatment before surgery and had been characterized in detail in our previous work [see (45)], on the histological sections obtained from both tuberculoma walls and, in parallel, distant lung tissue parts that, pathomorphologically, corresponded to samples from the “Type II” and “Type III” groups, respectively. The collected data were not included in this work, as, in the absence of the *ex vivo* cell cultures, the *Mtb* loads remained unknown for these tissue samples, while acid-fast *Mtb* as well as LAM- and ESAT-6- or Rv2623-positive *Mtb*, solitary or as colonies, were found in macrophages on the histological sections for the TB lesions examined [see **Supplementary Figure 4** in (45)]. Notably, these data also confirmed the independence of macrophage polarization in human lungs from the anti-TB treatment of patients before surgery. Overall, further validation of these results using larger sample sizes from TB patients’ lungs is required.

Overall, our findings demonstrate the critical nature of tissue microenvironments in the regulation of the innate immune response and macrophage activation in the lungs of patients with active TB disease, where lung lesions range from immune-deficient (especially tuberculomas) to immune-vigorous with excessive inflammation (especially alveolar tissues). While HDT for TB shows promise for cytokine therapy development, current regimens fail to account for local immune response variations in patients’ lungs. Accordingly, anti-TB therapy with exogenous IFNγ and IL-12 has caused serious immune-related complications or limited efficacy in some TB patients, with detrimental effects on *Mtb* infection control (18–20, 29, 62, 100). Therefore, a comprehensive understanding of host immune responses to *Mtb* infection and the immune mechanisms driving TB progression in patients’ lungs is essential for developing new therapies or repurposing existing ones.

In general, our results attribute crucial role of cytokine signaling and the NF-*κ*B pathway in regulating the complex interaction and clinical behavior of *Mtb* and host cells in the patients’ lungs, when, with pulmonary TB disease progression, a disordered immune balance leads to the host’s inability to keep the infection under control.

## 5 Conclusion

The outcome of *Mtb* infection is closely associated with the strength of the host’s immune response in human lungs, where alveolar macrophages are the frontline cells controlling immune surveillance, defense, and regulation by recognizing and eliminating bacteria, releasing cytokines, and interacting with other immune cells to promote the pathogen clearance. In parallel, these cells represent a persistent reservoir of *Mtb* during TB progression. For a clearer understanding of macrophage immunobiology and host cells-*Mtb* interactions, the dynamics of the cytokine network activity and some polarizing stimuli, connected with the AhR and NF-*κ*B pathways, were analyzed in various lung tissues of patients with active TB disease. While each TB lesion had its unique infection trajectory and cytokine profile of macrophages, our study revealed the fundamental patterns of cytokine signaling activity, which were shaped by the local environmental milieu related to fibrosis severity, but not by the *Mtb* features or anti-TB treatment, in the lungs of the same patients and were repeated between different patients studied. The bacterial control, but not complete *Mtb* eradication, was associated with the NF-*κ*B-induced inflammation and co-expression of pro- and anti-inflammatory cytokines by smoker’s alveolar macrophages that exhibited a mixed M1/M2 polarization state in the TB lesions with local/minimal fibrosis. For the same patients, suppressed smoker’s macrophages that shared the M0-like polarization state without production of either M1 or M2 cytokines and inhibition of NF-*κ*B-mediated processes, but with AhR signaling activation, demonstrated significantly higher *Mtb* loads in tuberculoma walls and distant lung tissues with excessive/extensive fibrosis. Our findings demonstrate the complex and dynamic states of macrophage activation at different infection sites in the patients’ lungs and represent the immunologic basis of failed immunity during TB disease. This knowledge is critical in the design of immune-modulatory anti-TB therapeutic approaches that aim either to enhance the ability of the host immune system to eliminate *Mtb* or to limit the lung tissue damage associated with infection. Our data also indicate that immunological interventions involving strategic reprogramming of alveolar macrophage phenotypes should always be optimized while taking into account the specific properties of cells in the local lung microenvironment of TB patients, thereby emphasizing the need for further studies in this field.

## Data Availability

The original contributions presented in the study are included in the article/**Supplementary Material**. Further inquiries can be directed to the corresponding author.
