# Supplementary material for: Lesion-specific features of macrophage polarization contribute to Mycobacterium tuberculosis infection in the lungs of patients with pulmonary tuberculosis
Source: Front Immunol. 2026 Jun 12;17:1853352. doi: 10.3389/fimmu.2026.1853352 (PMC13303375; doi:10.3389/fimmu.2026.1853352)
Supplement: Supplementary file 1 [file SupplementaryFile1.docx]

Supplementary Material

# Supplementary Table 1. The characteristics of patients with pulmonary tuberculosis and the pathogen *M. tuberculosis* (*Mtb*) that infected their lungs

| Patients | Age (years) | Sex*^1^* | Smoking status (years, pack-years) | Drugs*^2^* before surgery | | Surgery*^3^* | | Lung tissue for *ex vivo* expansion of cells*^4^* | | *Mtb* | | | | | |
| --- | --- | --- | --- | --- | --- | --- | --- | --- | --- | --- | --- | --- | --- | --- | --- |
|  |  |  |  |  |  |  |  |  |  | Genotype family group*^5^* | | Viru-lence*^6^* | | Clinic isolate*^7^* | |
| 1 | 27 | M | 11, 11 | Z Pt Cap Pas Cs Of | | S6 LL | | distant part | | Beijing | | n.d. | | - | |
| 2 | 59 | M | 40, 40 | H R Z Pt Cap | | UL LL | | distant part | | Beijing | | n.d. | | - | |
| 3 | 36 | M | 20, 11 | H Z Pt Cap Pas Cs | | UL RL | | distant part | | non-Beijing | | n.d. | | - | |
| 4 | 33 | M | 15, 8 | H R Z E | | S1-2 RL | | distant part | | non-Beijing | | n.d. | | - | |
| 5 | 20 | M | 3, 6 | H R Z E Pt Cap Cs | | S1-3 LL | | distant part | | ND | | L | | - | |
| 10 | 45 | F | 20, 20 | Z E Pt Cap Pas Cs | | RL | | distant part | | Beijing | | H | | + | |
| 11 | 43 | M | 20, 10 | Z, Pt, Cap, Pas, Cs | | S1-2 RL | | distant part | | Beijing B0/W148 | | L | | + | |
| 12 | 51 | F | 0, 0 | Z Pt Cap Pas Cs | | UL RL | | distant part | | Beijing | | L | | - | |
| 13 | 59 | M | 30, 40 | H R Z Pt Pas | | S2 LL | | distant part | | ND | | L | | - | |
| 14 | 38 | M | 4, 1.5 | R Z E Pas | | S6 LL | | distant part | | Beijing | | L | | - | |
| 15 | 25 | M | 0, 0 | H R Z E | | S1-3 LL | | distant part | | Beijing | | L | | - | |
| 16 | 43 | M | 23, 27 | Z Pt Cap Pas Cs Of | | UL RL | | distant part | | Beijing | | L | | - | |
| 17 | 24 | F | 6, 3.5 | H Z E Rfd | | S1-2 LL | | distant part | | Beijing | | L | | - | |
| 18 | 28 | M | 0, 0 | H R Z E | | S1-2 LL | | distant part | | Beijing | | IM | | - | |
| 19 | 36 | M | 18, 18 | Z Pt Cap Pas Cs | | S1-2 RL | | distant part | | Beijing | | L | | - | |
| 20 | 33 | F | 18, 20 | H Z E Rfd | S6 RL | | distant part | | Beijing B0/W148 | | H | | + | |  |
| 21 | 33 | F | 13, 13 | Z E Pas Cs Of | S6 RL | | distant part | | ND | | L | | - | |  |
| 22 | 55 | M | 30, 15 | Z E Pas Cs K Of | S1-2 RL | | distant part & tuberculoma | | Beijing | | L | | - | |  |
| 23 | 28 | M | 2, 1 | H R Z E | S1-2 LL | | distant part & tuberculoma | | Beijing | | L | | - | |  |
| 24 | 63 | M | 43, 43 | Z Pt Cap Pas Cs Lfx | S1-2 RL | | distant part & tuberculoma | | Beijing | | L | | - | |  |
| 25 | 42 | M | 25, 37.5 | Z Pt Cap Pas Lfx | UL RL | | distant part & tuberculoma | | Beijing | | L | | - | |  |
| 26 | 57 | M | 17, 17 | Z Pt Pas Cs K Lfx | S1-2 RL | | distant part & tuberculoma | | Beijing | | L | | - | |  |
| 27 | 45 | M | 5, 5 | E Am Cs Lfx | S6 RL | | distant part & tuberculoma | | Beijing | | L | | - | |  |
| 28 | 58 | M | 40, 40 | H R Z E | S1-2 LL | | distant part & tuberculoma | | Beijing | | L | | - | |  |
| 29 | 54 | M | 30, 15 | H R Z E | S1-2 RL | | distant part & tuberculoma | | Beijing | | L | | - | |  |

*^1^*M, male; F, female.

*^2^*AK, aminoglycosides; Am, amikacin; Cap, capreomycin; Cs, cycloserine; E, ethambutol; Fq, fluoroquinolones; H, isoniazid; K, kanamycin; Lfx, levofloxacin; Of, ofloxacin; Pas, para-aminosalicylic acid; Pt, protionamide; R, rifampicin; Rfd, rifabutin; Z, pyrazinamide.

*^3^*S, segment; UL, upper lobe; LL, left lung; RL, right lung.

*^4^*Distant part – lung tissue far from macroscopic TB lesions (tuberculomas and cavities); tuberculoma – lung tissue from tuberculoma wall.

*^5^*ND, not determined.

*^6^*Characteristic in the guinea pig model of TB disease. Degree: L, low; IM, intermediate; H, high. n.d., not done.

*^7^Mtb* growth (colonies) on Lowenstein-Jensen medium. (+), is present; (-), is absent.

**Supplementary Figure 1**


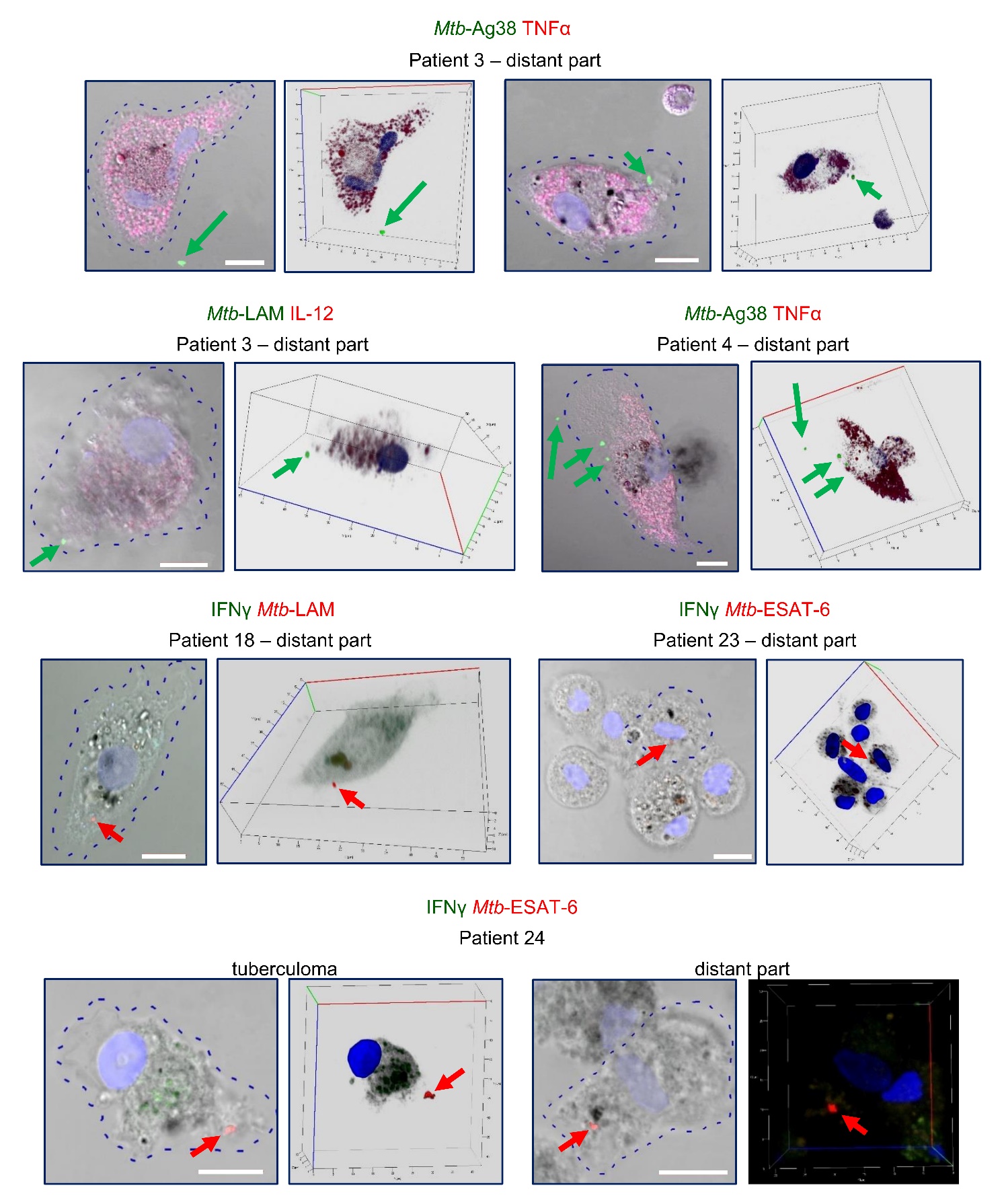


**Supplementary Figure 1.** Phase contrasted fluorescent images of macrophages help to define a localization of the pathogen in and/or outside the cytoplasm of cells – with or without cytokine production – after *ex vivo* culture for 16-18 hours. Representative confocal 3D and phase contrasted 2D merged fluorescent images (right and left panels, respectively) of the macrophages stained by antibodies reacting with human cytokines and *Mtb* antigens (green or red signals) are shown. Nuclei are stained by DAPI (blue signal). Short and long arrows (green or red) point to *Mtb* in and outside the cytoplasm of macrophages, respectively. To outline the cell contour, dotted blue lines are used. The scale bars are 10 μm each.

**Supplementary Figure 2**


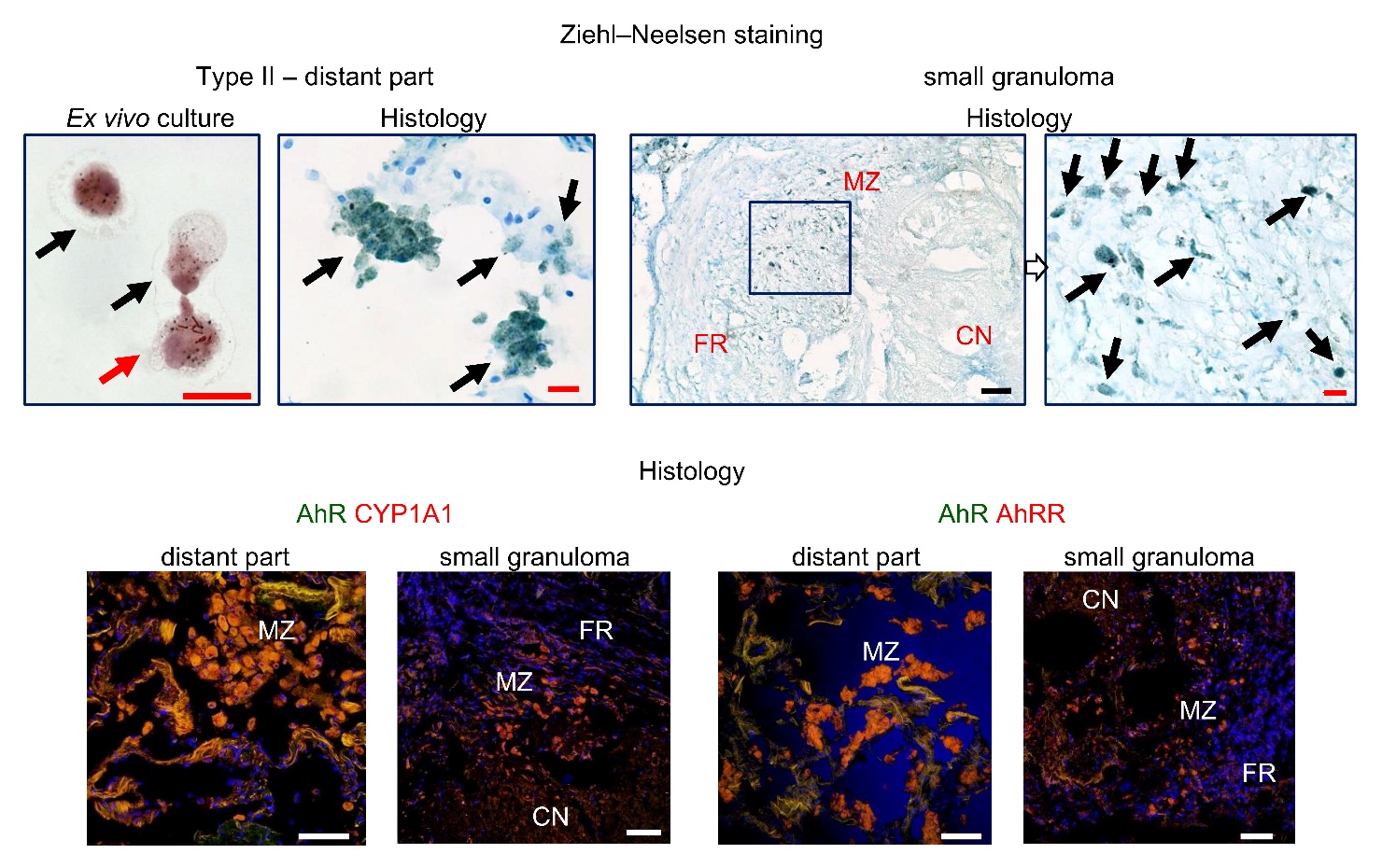


**Supplementary Figure 2.** Smoker's macrophages with activation of the AhR signaling pathway are observed not only within the airspace and interstitial areas of alveoli, but also in small granuloma in the same lung tissue sample of patient 14. Representative light and confocal merged fluorescent images of the macrophages stained by the Ziehl–Neelsen (ZN) method or antibodies reacting with AhR (green signal) and CYP1A1 or AhRR (red signal) and analyzed on the histological sections and, in parallel, only for ZN staining, after *ex vivo* culture for 18 hours are shown. Colocalization of the markers is yellow signal. Nuclei are stained by DAPI (blue signal). Close-up of the part of the ZN image with smoker's macrophages on the central-right panel is shown in the right panel. Red and black arrows point to smoker’s macrophages, as solitary or in clusters, with acid-fast *Mtb* in colony and without the pathogen in them, respectively. Collagen fibers are strongly autofluorescent (yellow signal) on histological immunofluorescent images. CN – caseous necrosis, FR – fibrotic region, MZ – macrophage-rich zone with the marker-positive cells. Red, black, and white scale bars are 20, 100, and 50 μm each, respectively.

**Supplementary Figure 3**


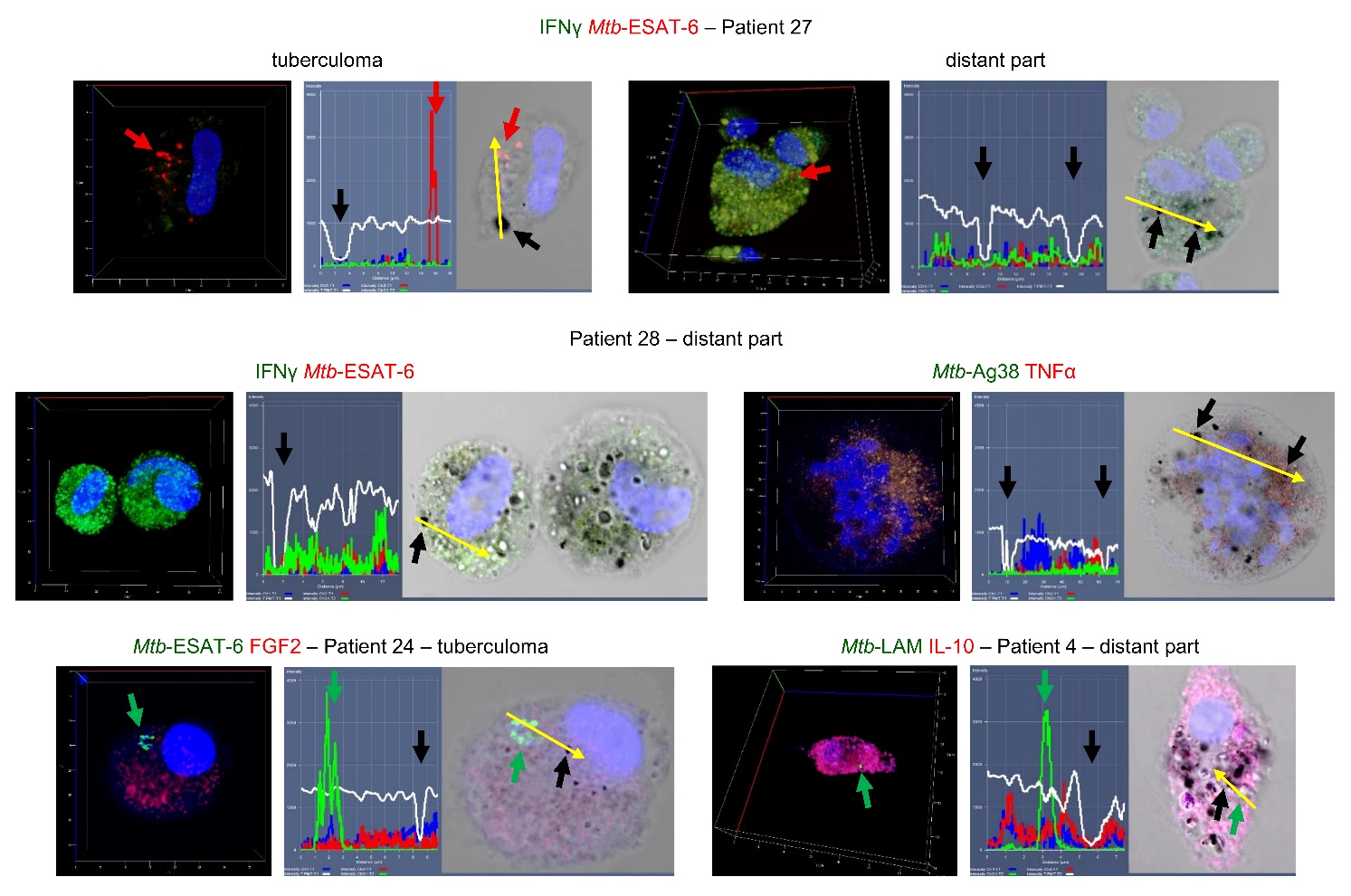


**Supplementary Figure 3.** Dense dark cytoplasmic inclusions do not demonstrate autofluorescent signals in the green and red channels on the confocal immunofluorescent images of the patients’ macrophages and multinucleate Langhans giant cell after *ex vivo* culture for 18 hours. Representative confocal 3D merged fluorescent images (left and central-right panels) and phase contrasted 2D merged fluorescent profile images (central-left and right panels) of the macrophages stained by antibodies reacting with human cytokines and *Mtb* antigens (green or red signals) are shown. Nuclei are stained by DAPI (blue signal). Yellow arrows point to the areas for constructing profile graphs. Black arrows point to the same dark cytoplasmic inclusions (solitary or in clusters) in the cytoplasm of cells and in the graphs of phase contrasted profile images. Green or red arrows point to *Mtb* (solitary or as colonies and cords) in all images.

**Supplementary Figure 4**


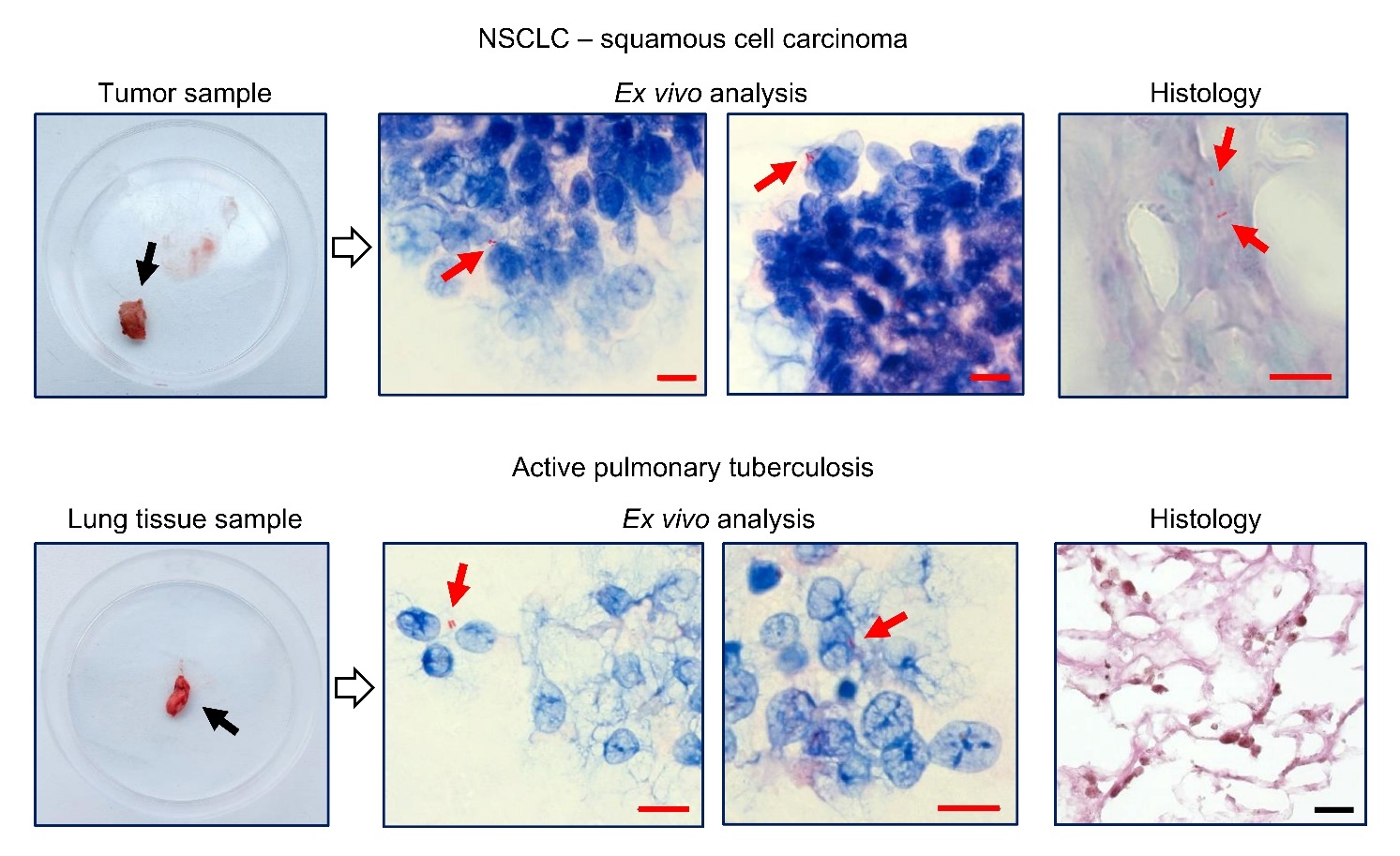


**Supplementary Figure 4.** Not only alveolar, but also tumor-associated macrophages with acid-fast *Mtb* in them are detected in the resected lung tissue (the lung part about 5 cm away from the tumor) and tumor samples, respectively, obtained from the same NSCLC patient (62-years-old male) with concurrent squamous cell carcinoma (Т1сN0М0(IА3)) and pulmonary TB, when the patient received a three-month course of anti-TB therapy with isoniazid, rifampicin, pyrazinamide, and ethambutol before surgery. Representative images of the cells stained by the Ziehl–Neelsen method and analyzed on the *ex vivo* cell preparations and, in parallel, histological sections are shown. Petri dishes with 5 cm in diameter. The surgical specimens are indicated by black arrows. Red arrows point to acid-fast *Mtb* in macrophages. Red and black scale bars are 10 and 20 μm each, respectively.
